# The inhibitory control of traveling waves in cortical networks

**DOI:** 10.1371/journal.pcbi.1010697

**Published:** 2023-09-05

**Authors:** Grishma Palkar, Jian-young Wu, Bard Ermentrout

**Affiliations:** 1 Department of Mechanical Engineering and Material Science, University of Pittsburgh, Pittsburgh, Pennsylvania, United States of America; 2 Department of Neuroscience, Georgetown University, Washington, DC, United States of America; 3 Department of Mathematics, University of Pittsburgh, Pittsburgh, Pennsylvania, United States of America; Western University, CANADA

## Abstract

Propagating waves of activity can be evoked and can occur spontaneously *in vivo* and *in vitro* in cerebral cortex. These waves are thought to be instrumental in the propagation of information across cortical regions and as a means to modulate the sensitivity of neurons to subsequent stimuli. In normal tissue, the waves are sparse and tightly controlled by inhibition and other negative feedback processes. However, alterations of this balance between excitation and inhibition can lead to pathological behavior such as seizure-type dynamics (with low inhibition) or failure to propagate (with high inhibition). We develop a spiking one-dimensional network of neurons to explore the reliability and control of evoked waves and compare this to a cortical slice preparation where the excitability can be pharmacologically manipulated. We show that the waves enhance sensitivity of the cortical network to stimuli in specific spatial and temporal ways. To gain further insight into the mechanisms of propagation and transitions to pathological behavior, we derive a mean-field model for the synaptic activity. We analyze the mean-field model and a piece-wise constant approximation of it and study the stability of the propagating waves as spatial and temporal properties of the inhibition are altered. We show that that the transition to seizure-like activity is gradual but that the loss of propagation is abrupt and can occur via either the loss of existence of the wave or through a loss of stability leading to complex patterns of propagation.

## Author summary

Stimuli and other aspects of neuronal activity are carried across areas in the brain through the concerted activity of recurrently connected neurons. The activity is controlled through negative feedback from both inhibitory neurons and intrinsic currents in the excitatory neurons. Evoked activity often appears in the form of a traveling pulse of activity. In this paper we study the speed, magnitude, and other properties of these waves as various aspects of the negative feedback are altered through both computational modeling and manipulations of a slice of cortex. Inhibition enables information to be readily transmitted across distances without the neural activity blowing up into a seizure-like state.

## Introduction

Recordings in different cortical regions and layers during sensory stimulation show that the response often manifests as a traveling wave [1–6] even though the stimulus is localized. In these waves, groups of neurons fire transiently in succession and then fall into a depressed (refractory) state before returning to rest. Evoked waves are distinct from the waves that are seen in local field potentials of humans and animals which appear as spatio-temporal phase gradients in ongoing activity [1, 7]. Evoked waves are more akin to traveling action potentials in excitable media while the latter are more like the waves that arise from coupled oscillators [8].

When waves are evoked by an external stimulus, the firing of excitatory cells is sparse (a characteristic of so-called balanced networks of excitation and inhibition [9]), yet the wave manages to propagate over centimeters of tissue. In contrast, when inhibition is pharmacologically blocked, particularly in slice preparations, the waves involve many excitatory neurons, have much broader profile (when imaged with voltage sensitive dyes) and travel faster [5, 10]. These reduced-inhibition waves have been implicated in seizure propagation models [11–13]. This suggests that the sparsity and degree of participation of excitatory cells in the propagation of evoked waves is controlled by negative feedback. (See also [14].) There are at least two sources to this negative feedback: recurrent inhibition and activity-dependent adaptation in the excitatory cells. Thus, our goal in this paper is to explore how these two effects work to control the macroscopic properties of evoked waves.

While direct evidence of the functional importance of these evoked waves in sensory processing has not been found, a number of hypotheses have been suggested [1, 8]. Ferezou et al [6] showed that in awake behaving mice, a solitary whisker flick evoked large-amplitude propagating sensory activity across the barrels. Similarly, [15] found that the population response to evoked visual stimuli in awake monkeys was a propagating wave. They suggest that these waves can modulate the excitability of the cortex in such a way as to affect processing of future stimuli. More recently, [16] have suggested that propagating waves in motor cortex can facilitate the initiation of movement and [17] have shown that spontaneous propagating waves can affect perception in awake monkeys. Because of their possible roles in modulating cortical excitability in a spatially and temporally precise manner, it is important to better understand the cellular mechanisms that control these waves.

The earliest computational models for traveling waves (TWs) in neural media can be found in the the work of Wilson and Cowan [18] and Amari [19] who looked at dynamics in neural fields. Later models of TWs using Hodgkin-Huxley type spiking models with distance dependent coupling explored waves in thalamus [20] and disinhibited cortex [21]. Ermentrout and others [22, 23] provided some early attempts for the analysis of TWs in spiking models. Most of the analytic work on TWs has been done for neural field or firing rate models, where the activity of populations of neurons is modeled rather than individual cells. Pinto & Ermentrout [11] analyzed a model which had a population of excitatory cells and a linear adaptation variable using a combination of analytic methods and singular perturbation theory. There have been many extensions of this work with comprehensive reviews found in [24, 25]. In addition to the existence of TWs in neural field models, their stability has been an important area of study. For piece-wise linear (PWL) models, Coombes and his collaborators have been instrumental in developing a toolkit for stability analysis using the so-called Evans function formulation [26, 27].

All of the above mentioned computational papers focused on either recurrent inhibition, spike frequency adaptation, or synaptic depression as means of preventing run away excitation. Only recently have models been suggested which involve multiple types of negative feedback. Gonzalez et al [13] analyze a system of excitatory and inhibitory rate models where the excitatory population includes adaptation. In their model, propagation occurs only when the inhibition is weak and slow. In the discussion, we will examine in more detail differences between their model and ours.

In this paper, we are interested in studying the effects of recurrent inhibition and spike frequency adaptation on the properties of evoked waves. We first show stimulus evoked waves in both *in vivo* and *in vitro* preparations form rat cortex with intact inhibition and by gradually blocking inhibition, we look at properties such as the magnitude and speed of the waves. We propose a spiking network of excitatory and inhibitory neurons based on the quadratic-integrate-and fire model (QIF) and use this to study propagation as the inhibition is varied. We also show how a single evoked wave can affect subsequent stimuli in a timing-dependent manner. Next, we develop a simple firing rate model based on the known firing rate properties of the QIF model that is similar to a Wilson-Cowan type model. We use numerical continuation and simulations to study the effects of recurrent inhibition and adaptation on the velocity of evoked TWs. We find some interesting instabilities of the waves as the footprint of the inhibition increases allowing us to connect the failure to propagate with transition to stationary spatial patterns such as bump attractors and Turing patterns. Finally, we replace the smooth nonlinearities of the neural field model with step functions which enables us to obtain analytic results on the properties of the waves and their stability. We conclude with some comparisons of our modeling to previous work and a brief discussion of the implications of this work on cortical computations.

## Results

### Experimental results

**Fig 1:**
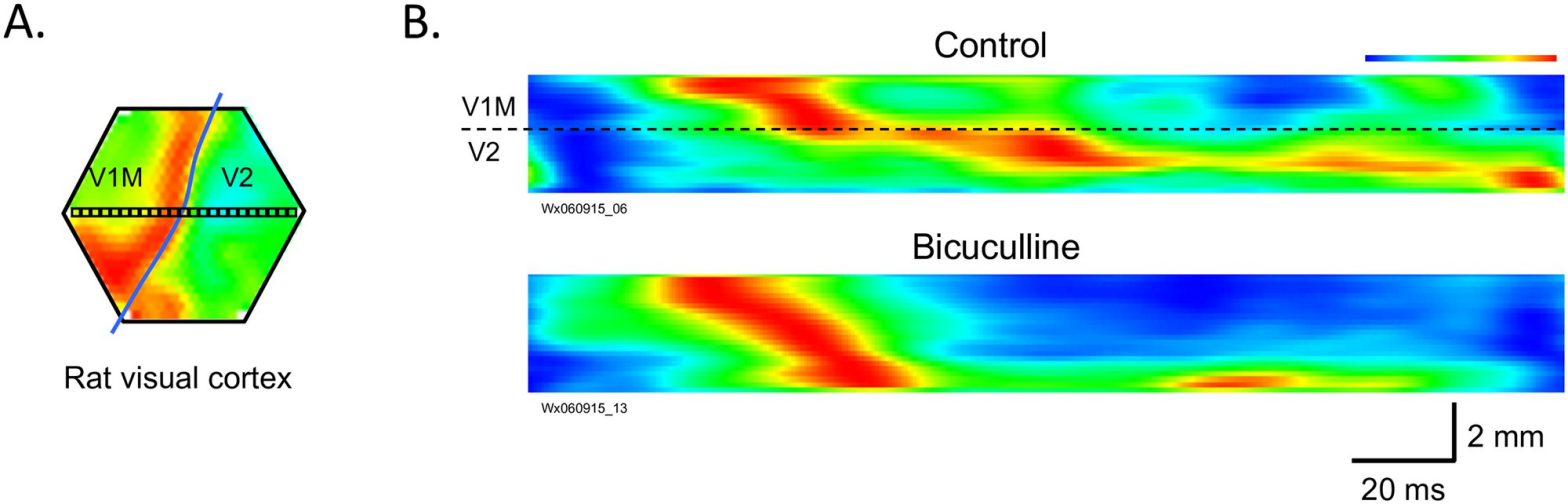
(A) Rat visual cortex was imaged by a voltage sensitive dye. Excitation waves are represented by a pseudo-colors map, where red (blue) represents maximal (minimal) excitation of the cortex. The blue line marks the border between primary (V1M) and secondary(V2) areas of the visual cortex. The voltage dye signals were detected by 464 optical detectors over the field of view (approximately 5 mm in diameter). Signals from a line of detectors (small boxes) were selected to make the space-time plot in B. (B) The horizontal and vertical axes of the graphs represent time (msec) and distance (mm) respectively. The top papen shows the stimulus evoked wave in normal inhibition, while in the bottom panel, inhibition has been partially blocked. (Modified from [28]).

We begin this paper with experimental demonstrations of evoked traveling waves in an anesthetized rat and then in a cortical slice preparation where we can better observe and manipulate the evoked wave. In Fig 1 we show an example of a wave evoked by a visual stimulus (moving gradient) in the visual cortex of an anesthetized rat (adapted from Fig 6 in [28]). The wave originates in primary visual cortex (V1M) and propagates to other visual areas (V2) (panels A,B) but slows down dramatically after crossing the V1M/V2 border. This slowing down can be eliminated by topical application of 3*μ*M bicuculline leading to a large increase in the velocity. In an earlier paper [29], we modeled the mechanisms of compression across the boundaries of the two cortical regions.

**Fig 2:**
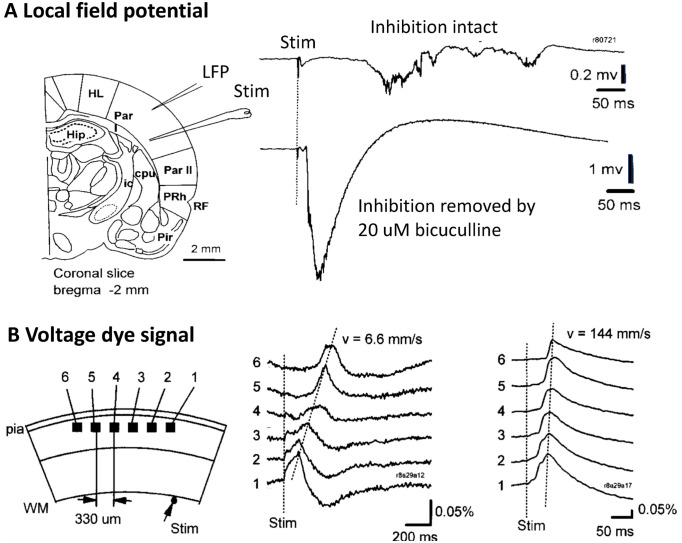
Evoked cortical activity and propagation velocity in a rat cortical slice. A. Neuronal activity measured by local field potential electrode. B. Propagating velocity measured by voltage-sensitive dye and optical recording. (Modified from [30]).

To further quantify the effects of recurrent inhibition in cortical networks, we recorded evoked activity in an acute cortical slice preparation (see Materials & methods for details). Stimulation is delivered to the cortical tissue by a glass pipette with a 10*μ*m tip opening. It consists of a voltage pulse of 1-10V, and width of 0.05 ms. With the pipette tip resistance of ∼ 50*K*Ω the current flowing to the tissue is 20-200 nA. This stimulation induces a local pulse of population activity propagating slowly though the cortex (Fig 2A, right top trace). After removing inhibition with 20 *μ*M of bicuculline, the same stimulation induced a large and fast pulse of activity (Fig 2A, bottom trace). Note that removing inhibition also largely reduced the latency of the evoked response. The latency included the time for the activity spreading from stimulation site to the recording site, and so the reduced latency suggested a faster propagating speed after removal of inhibition. The propagation velocity was further examined with multiple recording sites (Fig 2B). In this experiment we used voltage-sensitive dye to convert membrane potentials to a light intensity signal. Each optical detector receives light from thousands of cortical neurons, and the optical signal is thus an integration of membrane potential change of all the neurons under one detector. We found that removing inhibition increased the propagating speed of the cortical activity (> 20×). Our results have been verified by more than 100 slices since [30] (for example, see [28, 31, 32]). This example clearly shows several properties of the wave in normal and disinhibited slices. First, the response is substantially greater in both magnitude and duration when inhibition is removed. Further the wave shape is more reliable during the propagation event and far less noisy. Lastly, the propagation velocity increased as the inhibition was decreased.

### Spiking model

**Fig 3:**
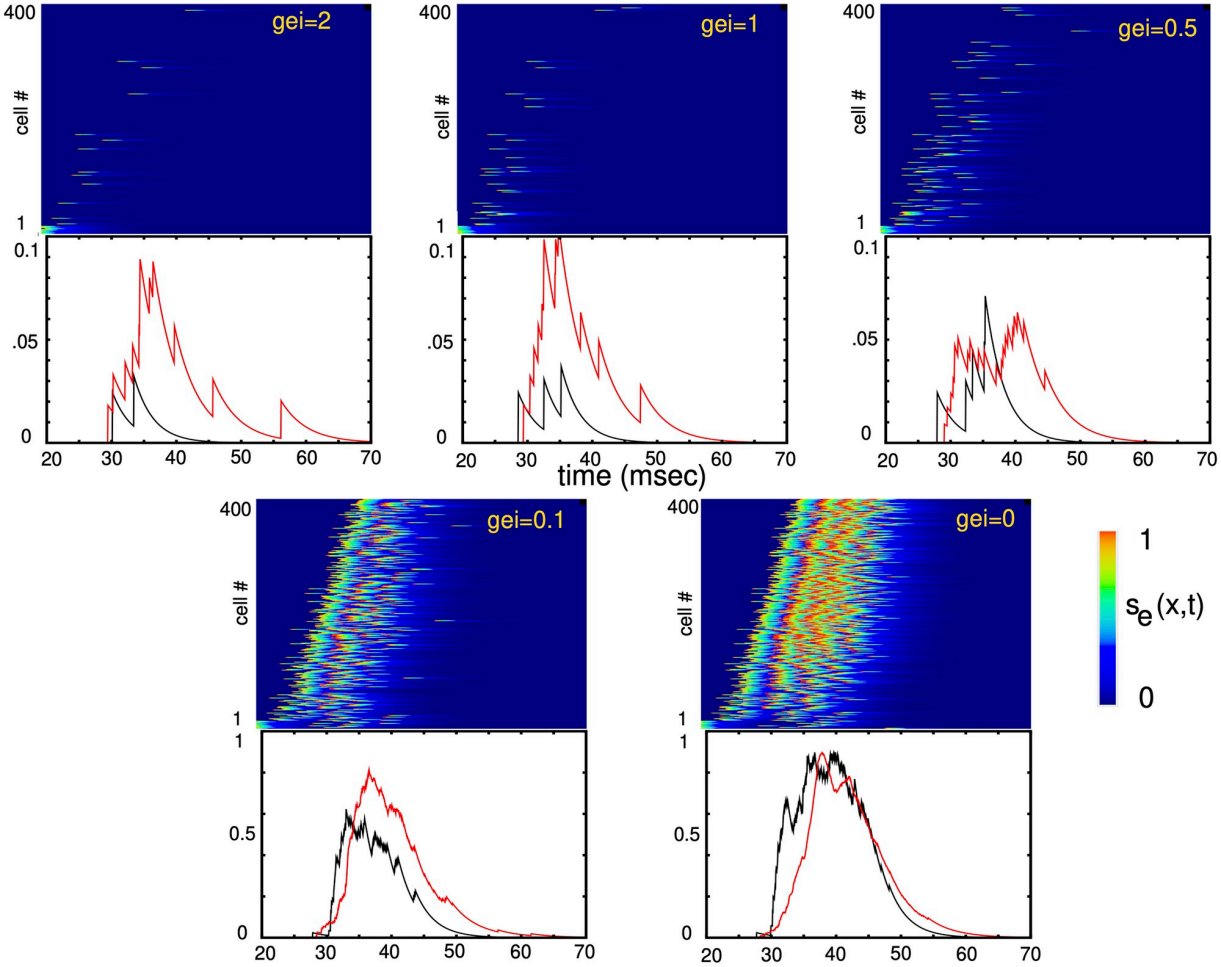
Traveling waves generated in the spiking models are controlled through the strength of feedback inhibition to the excitatory cells, *g*_*ei*_. Plots of the synaptic activity of each of the 400 excitatory cells are plotted in the top of each panel; below are the summed synaptic activity of a cluster of 40 excitatory (black) and 10 inhibitory cells (red) in the middle of the medium (excitatory cells 180-220; inhibitory cells 45-55). Inhibitory output has been scaled down for clarity.

To begin our computational analysis of the inhibitory control of TWs, we developed a spiking model that consisted of 400 excitatory and 80 inhibitory theta-neurons distributed on a one-dimensional line 0 < *x* < 1 with random distance-dependent connections (see Methods for details). The position of the *j*^*th*^ excitatory cell is *x* = *j*/400 and of the *j*^*th*^ inhibitory cell is *x* = *j*/80. Fig 3 shows plots of *s*_*e*_(*j*), for a particular network of 400 excitatory cells. At *t* = 5msec, a brief pulse of current is applied to the first 20 excitatory cells and evokes a wave. When the inhibition is high, only a small fraction of the excitatory cells participate in the wave, where as nearly all the inhibitory cells do. The wave takes about 20 msec to traverse the domain. As the recurrent inhibition (*g*_*ei*_) decreases, the number of excitatory cells participating in the wave increases dramatically, with nearly 100% participation once *g*_*ei*_ falls below about 0.25. There is a small increase in velocity, but it is not as dramatic as shown in Fig 2B. Increasing *g*_*ei*_ beyond 2 does little to affect the speed or participation. In addition to the raster plots, we also show the summed synaptic activity of the excitatory and inhibitory cells in the middle of the network. There is more than an order of magnitude increase in the excitatory amplitude as *g*_*ei*_ decreases.

**Fig 4:**
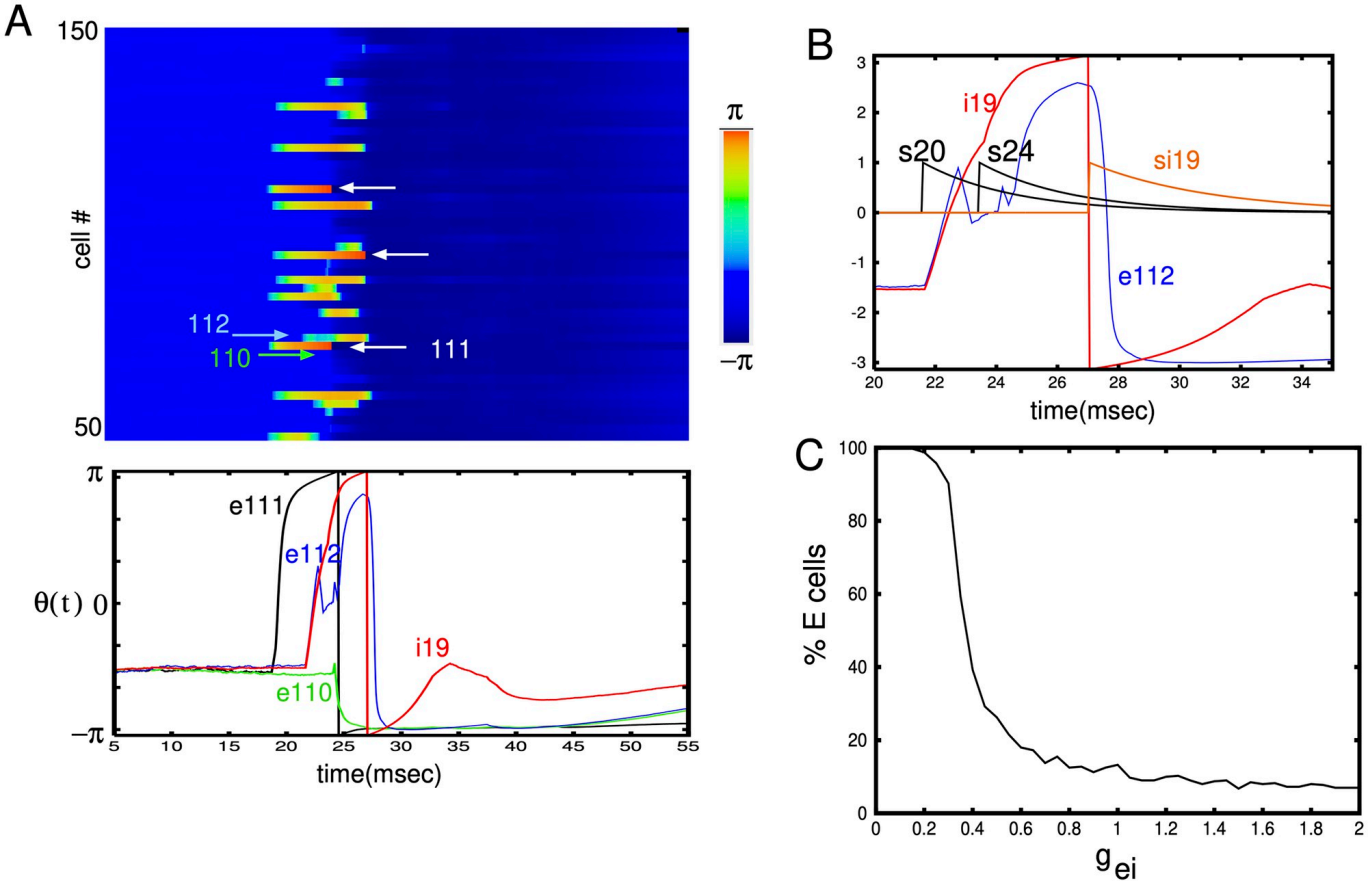
(A) Subthreshold dynamics (*θ*_*j*_(*t*)) for *g*_*ei*_ = 1 for the simulation shown in Fig 3. Cells that reached *π* (and fired) are indicated by the white arrows in the top panel. In the lower panel, time series of excitatory cells #110,#111,#112 and inhibitory cell #i19. (B) Expanded view showing how inhibition from cell #i19 blocks the ability of excitatory cell # e112 to generate a spike. The synapse from excitatory cell # 20 initiates both cells # i19 and # e112 but a second input to # i19 (s24) accelerates the spiking of # i19 allowing it to block # e112. (C) Fraction of excitatory cells participating in the wave as a function of *g*_*ei*_.

Fig 4A shows the subthreshold activity of a subset of the excitatory cells in the network for *g*_*ei*_ = 1. Recalling that the network is comprised of theta neurons where spiking occurse when, *θ*_*j*_(*t*) = *π*, we see that only 3 of the 100 cells ever spike although a much larger fraction of them are depolarized (warmer colors). The lower panel shows the time-traces of three excitatory cells (e110,e111,e112) as well as an inhibitory cell (i19) that is nearby. (The position of the excitatory cells is *x* ≈ 0.27 and of the inhibitory cell is *x* = 0.23.). Panel B in the figure shows an expanded view of e112 and i19 as well as the synaptic activity of e20,e24, and i19 in order to illustrate how the inhibition renders the excitatory participation sparse. Synapse se20 causes both cells e112 and i19 to begin to fire. A subsequent excitatory input into i19 (se24) accelerates the firing of i19 which then suppresses e112 so that it does not fire. In Panel C, we see how this sparsity is controlled by the inhibitory to excitatory strength. Because of the large number of excitatory to inhibitory synapses in our model, the inhibitory cells have a high probability of firing and thus keep the fraction of excitatory cells that participate to a low number as suggested in [14, 33]. Thus, the main ability to control excitation comes from the feedback inhibition which is determined by *g*_*ei*_. Once the strength of this falls below about 0.2 almost all excitatory cells beacome involved in the wave and it is equivalent to seizure-like activity where a given excitatory cell will fire a burst of activity. This activity is suppressed only by the spike-frequency adaptation.

**Fig 5:**
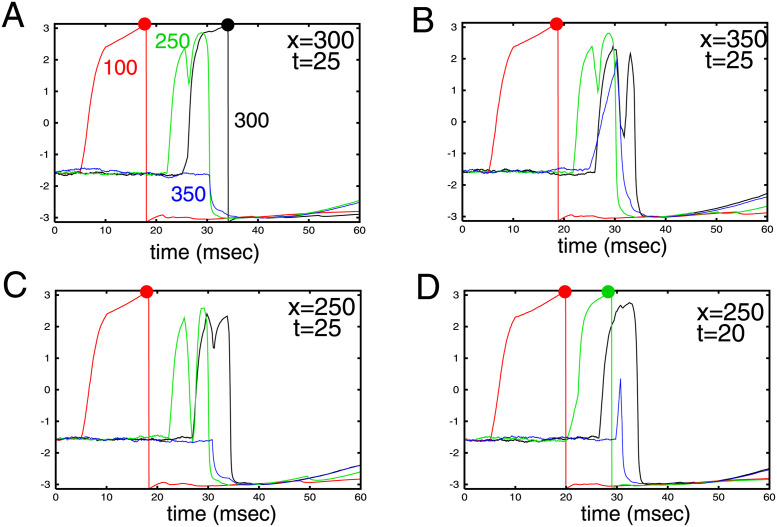
Waves can prime the network to selectively respond to subthreshold stimuli. Stimulus is a square pulse lasting 5 msec occuring at time *t* and width of 10 cells centered at position *x*. Images show cell #100 spiking which initiates the wave. Labels next to each curve are the transformed potentials of the corresponding excitatory cell. (A) stimulus at *t* = 25, *x* = 300 evokes spike in cell 300, but not in 250,350; (B) when the stimulus is shifted to *x* = 350, cell 350 is depolarized but fails to fire as it received no extra depolarization from the wave; (C) stimulus at *x* = 250 comes at the wrong time for cell 250; (D) stimulus time is shifted to *t* = 20 and causes cell 250 to spike.

As should be clear from Fig 4, some cells are depolarized due to the evoked wave, but not sufficiently to evoke a reponse. However, this depolarization can prime those cells or nearby cells to an appropriately timed stimulus. Davis et al [17] have shown that traveling waves can gate the perception of later stimuli. Here, we illustrate this effect in the spiking model by first evoking a wave and then providing a brief spatially localized stimulus at differing times and locations to cells that did not spike. Fig 5 shows stimuli to three different cells at different times after a wave is evoked (cell 100). In panel A, an appropriately timed stimulus to cell 300 evokes an action potential (black dot) about 7 msec after the stimulus onset. In the second panel, the same stimulus is applied to cell 350, but fails to evoke a spike namely because the wave did not cause any depolarization of this particular cell. In panel C, cell 250 (upstream from cell 300) is stimulated at *t* = 25 but because the simulus is late, it does not cause cell 250 to spike. Panel D shows that a slightly earlier stimulus evokes a spike in cell 250. The wave sets a spatial and temporal bias allowing for a direction sensitive sensitivity of stimuli that occur subsequently to the first stimulus. Ferezou et al [6] speculate that evoked waves in the whisker barrels of awake mice can facilitate multi-whisker integration. Here we have demonstrated such a synergestic effect in a spiking model.

**Fig 6:**
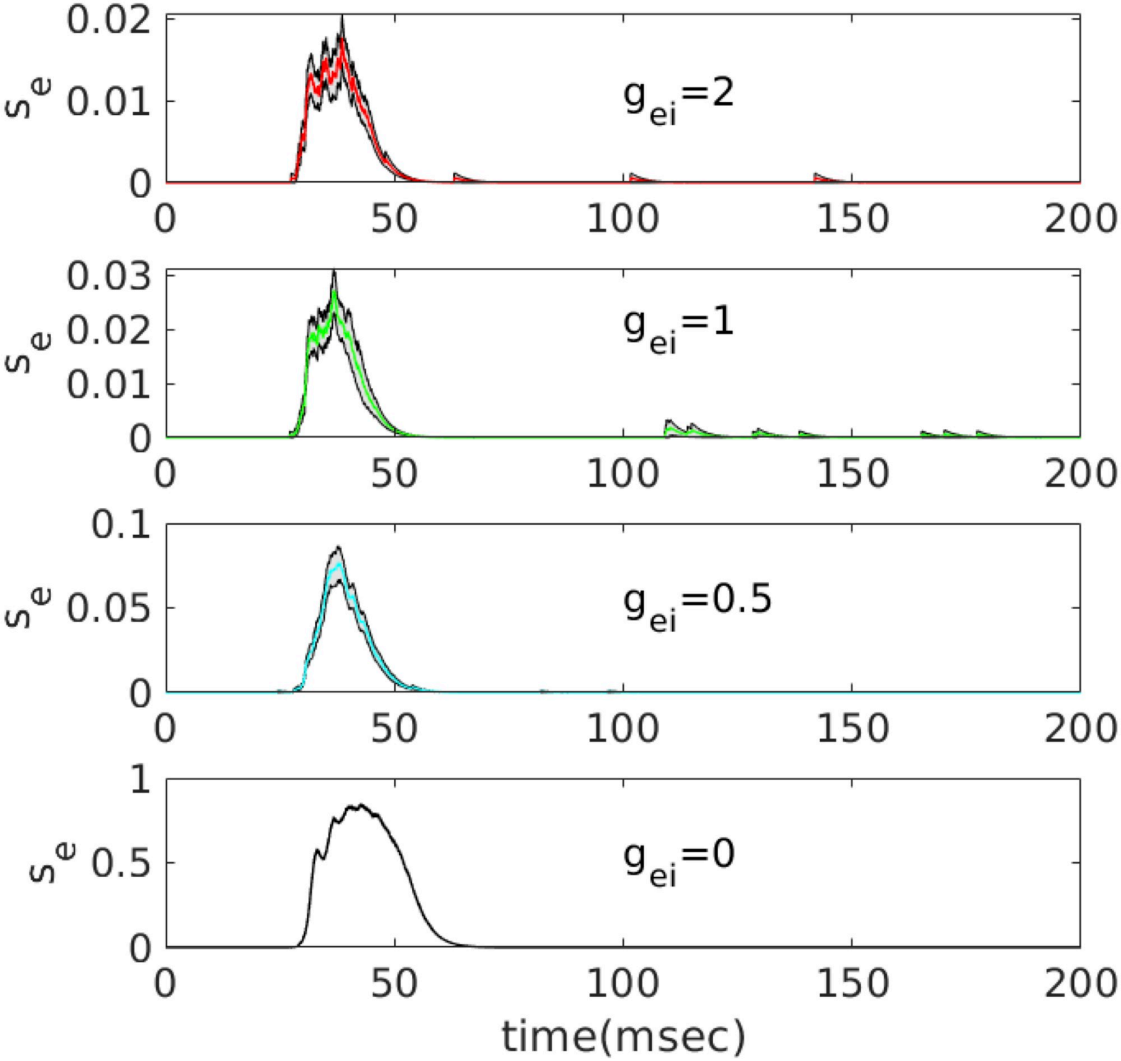
Behavior of the summed excitatory synaptic output over cells 180-220 for different values of *g*_*ei*_. In each plot, we regenerate a new connectivity matrix with all parameters the same and average over 40 such trials. Means are colored and the standard error is outlined in black.

**Fig 7:**
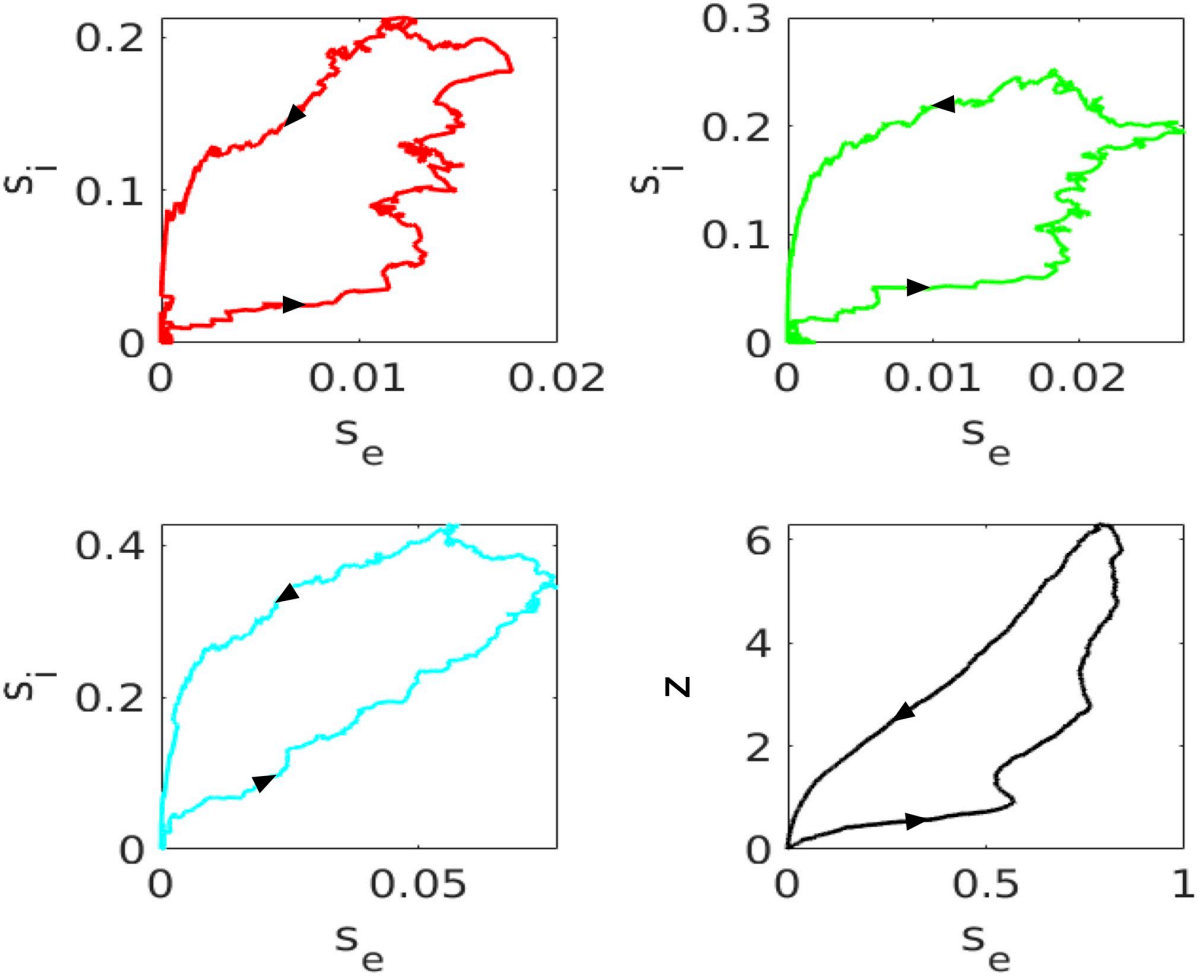
Projection of the excitatory activity from Fig 6 and the summed activity of inhibitory cells 35-45 over 40 repetitions of the simulation. Color code as in previous figure. In the lower right panel, we also plot the projection of the averaged value of the adaptation variable.

In preparation for our mean field analysis, we look at the time series for the synaptic activation of excitatory neurons (Fig 6) as well as a phase-plane projection of the excitatory and inhibitory synaptic dynamics (Fig 7) as we vary *g*_*ei*_. We run a simulation of the spiking model 40 times, each time *regenerating a new connectivity matrix* with the same statistics as the fixed network presented in Fig 3. We plot

$$\begin{array}{ccc} s_{e} & = & \frac{1}{40}\sum_{j=180}^{220}s_{e,j}\\ z_{e} & = & \frac{1}{40}\sum_{j=180}^{220}z_{e,j}\\ s_{i} & = & \frac{1}{10}\sum_{j=35}^{45}s_{i,j}, \end{array}$$

averaged over 40 trials. As expected, both the mean *s*_*e*_ and *s*_*i*_ increase a great deal as *g*_*ei*_ decreases. Since the adaptation, *z*_*j*_ matters only when there is very low *g*_*ei*_, we also show a phase-plane projection of (*s*_*e*_, *z*_*e*_) in the final panel of Fig 7. The magnitude is not as large as *s*_*i*_ but, because the decay of adaptation is quite slow compared to the inhibition, *z*_*e*_ persists long after the excitation is gone.

### Mean field reduction

The spiking model has the advantage that it can directly connect the behavior of individual neurons in sparsely connected random networks to the dynamics of the travelling waves. However, due to the stochasticity both in the coupling matrices and the applied background random noise, it is difficult to study the properties of the waves as multiple sets of parameters are varied. Furthermore, questions about how the existence of the waves is lost and whether they are stable cannot be addressed with the spiking model. For this reason, we will instead study a simplified *mean field* model that is based on a quasi-steady state approximation (see e.g., [34]). Consider a single spiking neuron with noise:

$$\begin{array}{c} C_{m}\frac{dV}{dt}=g_{L}\frac{\left(V-E_{L}\right)\left(V-E_{T}\right)}{E_{T}-E_{L}}+I+\hat{\zeta }\Xi \left(t\right) \end{array}$$ (1)

where *I* represents all the inputs to the neuron including the synaptic inputs, adaptation (for the excitatory cells), as well as the stimulus and any applied currents, and Ξ(*t*) is Gaussian white noise with magnitude $\hat{\zeta }$. In our simulations of the spiking model, we converted this quadratic differential equation to the theta model, but, here, we keep it here in the unconverted form as the calculations are easier. We emphasize that the two formulations (QIF/theta model) are equivalent. The time to spike is the time it takes for *V* to go from −∞ to +∞ (equivalent to *θ* going from −*π* to +*π*). We suppose that the noise, $\hat{\zeta }$ is zero. Then for *I* > I* = *g*_*L*_(*E*_*T*_ − *E*_*L*_)/4, we can compute the time it takes (the period) for *V*(*t*) to go from −∞ to +∞ by integrating Eq (1). Inverting this, we get an expression for the frequency

$$\omega \left(I\right)=\frac{1}{\pi }\sqrt{\left(\frac{g_{L}{\left[I-I^{*}\right]}_{+}}{c_{m}^{2}\left(E_{T}-E_{L}\right)}\right)}\equiv \frac{1}{\pi }\sqrt{\mu }$$

where [*I*]_+_ is the positive part of *I*. With noise, there can be firing when *μ* < 0, so we approximate the effects of noise by replacing the deterministic rate, *ω* with our nonlinear gain function:

$$\begin{array}{c} F\left(I,\zeta \right)=\frac{1}{\pi }[\frac{1}{2}\left(\mu \left(I\right)+\sqrt{\mu {\left(I\right)}^{2}+\zeta^{2}}\right){]}^{1/2} \end{array}$$ (2)

where

$$\mu \left(I\right)=\frac{g_{L}\left(I-I^{*}\right)}{c_{m}^{2}\left(E_{T}-E_{L}\right)},\;I^{*}=g_{L}\left(E_{T}-E_{L}\right)/4.$$

When *ζ* = 0, we recover the deterministic rate. Note that *F* is defined and nonzero for *I* < I*. This approximation is not arbitrary and arises in the rigorous reduction of the QIF when there is heterogeneity (see Eq. 5 in [35]). With the firing rate in hand, we can now write down a simple Wilson-Cowan like mean field model. The synapses in our spiking model obey:

$$\frac{ds}{dt}=-s/\tau_{s}+S\left(t\right)$$

where *S*(*t*) = ∑_*j*_ *δ*(*t* − *t*_*j*_) and *t*_*j*_ are the times that presynaptic neuron fires. The average *S*(*t*) is just the instantaneous firing rate, Eq (2) so that we obtain the mean field equation for *s*_*e*,*i*_:

$$\begin{array}{c} \frac{ds_{e,i}}{dt}=-s_{e,i}/\tau_{e,s}+F\left(I_{i,e}^{tot},\zeta_{i,e}\right) \end{array}$$ (3)

where $I_{i,e}^{tot}$ is the total current input into the inhibitory (i) or excitatory (e) populations. Similar to the synapses, that adaptation can be approximated by

$$\begin{array}{c} \frac{dz}{dt}=-z/\tau_{z}+F\left(I_{e}^{tot},\zeta_{e}\right). \end{array}$$ (4)

Before introducing the spatial model, we turn to the local dynamics of the EIZ network.

#### Local kinetics

**Fig 8:**
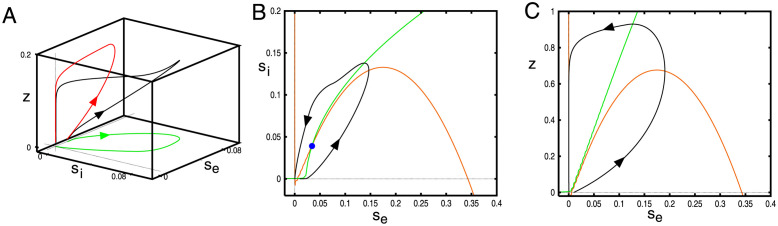
Excitability of the local equations (6). (A) Trajectory in (*s*_*e*_, *s*_*i*_, *z*) for *g*_*ei*_ = 2, *g*_*ad*_ = 0.25 with initial data (0.015, 0, 0). (B) (*s*_*e*_, *s*_*i*_)−plane when *g*_*ad*_ = 0, *g*_*ei*_ = 2 showing the excitatory (red) and inhibitory (green) nullclines (B) (*s*_*e*_, *z*)−plane when *g*_*ad*_ = 0.25, *g*_*ei*_ = 0 showing the excitatory (red) and adaptation (green) nullclines.

The spiking simulations suggest a pulse of activity propagates across the medium. The hallmark of pulse waves is an underlying excitable medium. Thus, we focus on the excitability of the local (*s*_*e*_, *s*_*i*_, *z*) system. With two exceptions, we will use the same parameters for the mean field model as we used in the spiking model. In the spiking model, we chose *g*_*ee*_ = 0.15, but for the mean field models we increase this to *g*_*ee*_ = 1. We also reduce *g*_*ad*_ = 1 to *g*_*ad*_ = 0.25, although we will vary this parameter later in the paper. From equations Eqs (3) and (4) we have:

$$\begin{array}{ccc} \frac{ds_{e}}{dt} & = & -s_{e}/\tau_{e}+F\left(g_{ee}\gamma_{e}s_{e}-g_{ei}\gamma_{i}s_{i}-g_{ad}\gamma_{z}z,d_{e}\right)\\ \frac{ds_{i}}{dt} & = & -s_{i}/\tau_{i}+F\left(g_{ie}\gamma_{e}s_{e}-g_{ii}\gamma_{i}s_{i},d_{i}\right)\\ \frac{dz}{dt} & = & -z/\tau_{z}+F\left(g_{ee}\gamma_{i}s_{e}-g_{ei}\gamma_{i}s_{i}-g_{ad}\gamma_{z}z,d_{e}\right), \end{array}$$ (5)

where *γ*_*e*_ = *E*_*syn*_ − (*E*_*T*_ + *E*_*L*_)/2, *γ*_*i*_ = (*E*_*T*_ + *E*_*L*_)/2 − *I*_*syn*_, and *γ*_*z*_ = (*E*_*T*_ + *E*_*L*_)/2 − *E*_*K*_. (Note that we have defined the *γ*_*j*_ is such a way so that they are all positive so that in the signs of the currents reflect whether they contribute positive or negative effects to the firing rates.) Fig 8A depicts the behavior for this three-dimensional system when *s*_*e*_ is perturbed away from the rest state. The recurrent excitation causes an initial amplification of *s*_*e*_(*t*) before the inhibition and adaptation suppress it. We can better explore the nature of the excitability by setting either of *g*_*ei*_ or *g*_*ad*_ to zero leading to a planar system. In panel B (*g*_*ad*_ = 0), we see that the nullclines intersect in three places. There is a single stable equilibrium near (*s*_*e*_, *s*_*i*_) = (0, 0), a saddle point near (0, 0.005) and an unstable node (blue circle). We superimpose a trajectory starting at (0, 0.01) which decays back to the rest state after an excursion in the plane. Thus, the (*s*_*e*_, *s*_*i*_) subsystem is characterized by Class I excitability [36]. In panel C, we allow the adaptation (*g*_*ad*_ = 0.25) but set *g*_*ei*_ = 0 and depict a typical trajectory starting at *z* = 0 and *s*_*e*_ = 0.01. Here there is only one equilibrium point and it is stable. The (*s*_*e*_, *z*) subsystem is thus characterized by Class II excitability. As can be seen by comparing A to (B,C), the combined adaptation and inhibition has a strong effect on the amplitude of *s*_*e*_ The plots in panels B,C are qualitatively similar to the plots of the averaged spiking model shown in Fig 7. We note that if *g*_*ee*_ is too small then the cubic nature of the *s*_*e*_-nullcline disappears and there is no excitability. We additionally note that with *g*_*ad*_ ≈ 0, then when *g*_*ei*_ falls below a critical value, there will be an additional stable fixed point with *s*_*e*_ > 0.1 so that the local dynamics will not be excitable, but instead, it will be bistable.

In sum, the mean field local kinetics show the hallmarks of an excitable system so that we expect that there can be robust travelling pulse waves in the spatially connected system.

### Smooth spatial system

We turn our attention to the full mean-field spatial model:

$$\begin{array}{ccc} \frac{\partial s_{e}\left(x,t\right)}{\partial t} & = & -s_{e}\left(x,t\right)/\tau_{e}+F\left(g_{ee}\gamma_{e}S_{e}\left(x,t\right)-g_{ei}\gamma_{i}S_{i}\left(x,t\right)-g_{ad}\gamma_{z}z\left(x,t\right),d_{e}\right)\\ \frac{\partial s_{i}\left(x,t\right)}{\partial t} & = & -s_{i}\left(x,t\right)/\tau_{i}+F\left(g_{ie}\gamma_{e}S_{e}\left(x,t\right)-g_{ii}\gamma_{i}S_{i}\left(x,t\right),d_{i}\right)\\ \frac{\partial z(x,t)}{\partial t} & = & -z\left(x,t\right)/\tau_{e}+F\left(g_{ee}\gamma_{e}S_{e}\left(x,t\right)-g_{ei}\gamma_{i}S_{i}\left(x,t\right)-g_{ad}\gamma_{z}z\left(x,t\right),d_{e}\right), \end{array}$$ (6)

where

$$\begin{array}{ccc} S_{e}\left(x,t\right) & = & \frac{1}{\sigma_{e}}\int_{D}W\left(\left(x-y\right)/\sigma_{e}\right)s_{e}\left(y,t\right)\;dy\\ S_{i}\left(x,t\right) & = & \frac{1}{\sigma_{i}}\int_{D}W\left(\left(x-y\right)/\sigma_{i}\right)s_{i}\left(y,t\right)\;dy \end{array}$$

and *W*(*x*) is either the exponential, *W*_*E*_(*x*) = exp(−|*x*|)/2 or the Gaussian, $W_{G}\left(x\right)=\text{exp}\left(-x^{2}\right)/\sqrt{\pi }$ kernel. For purposes of analysis, *D*, the integration domain will be the real line, but for simulations, it will be a discretized finite domain. In what follows, we will fix all parameters except those that deal with the negative feedback, *g*_*ei*_, *g*_*ad*_ and the spread of inhibition, *σ*_*i*_ which has some very interesting effects on the stability of the waves.

**Fig 9:**
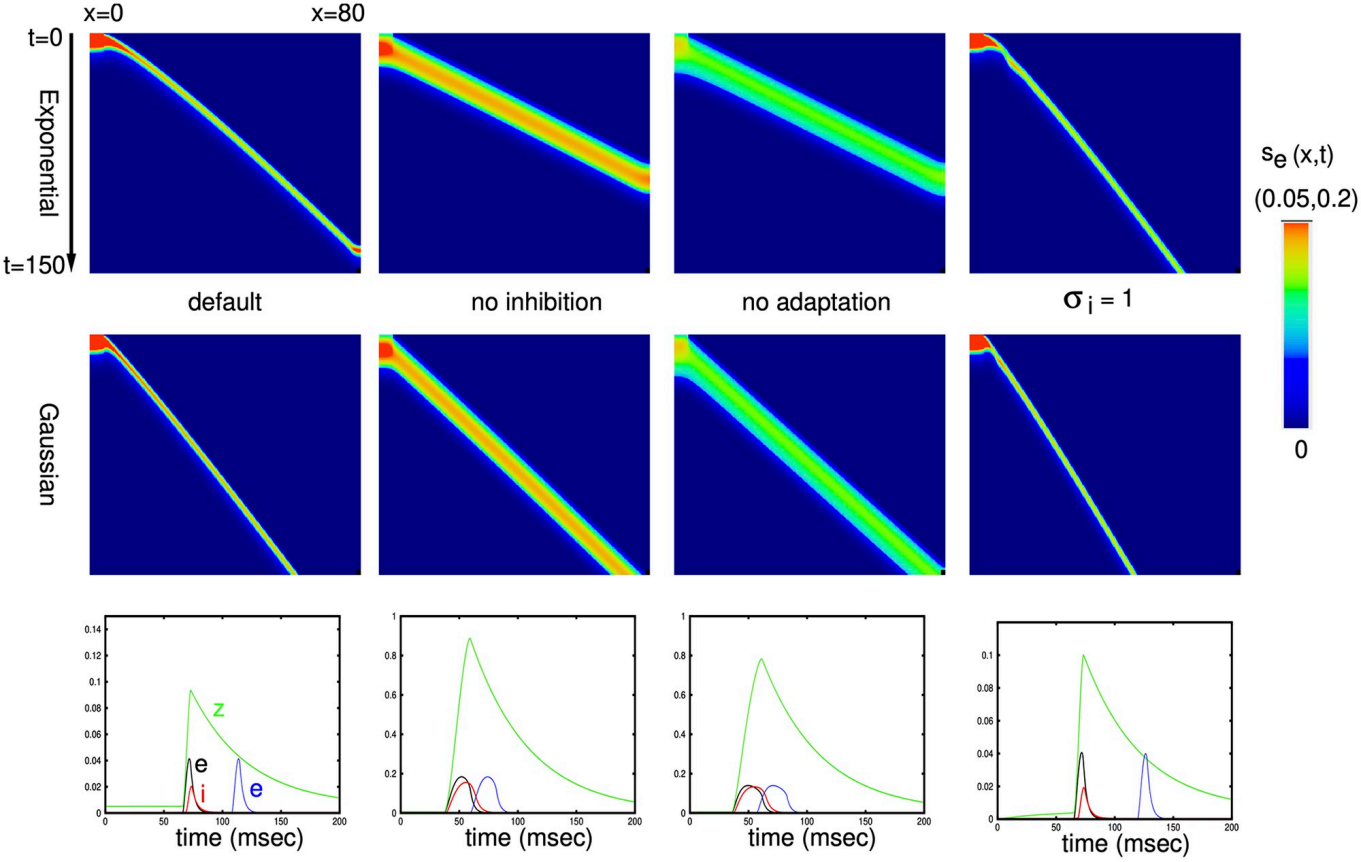
Plots of *s*_*e*_(*x*, *t*) for various parameters. Top row is the exponential kernel and second row is the Gaussian kernel. The default parameters are *g*_*ei*_ = 2, *g*_*ad*_ = 0.25, *σ*_*i*_ = 0.5. The color scale has a maximum of 0.05 for the outer two plots and 0.2 for middle two plots. Bottom row shows *s*_*e*_(40, *t*), *s*_*i*_(40, *t*), *z*(40, *t*) and *s*_*e*_(60, *t*) of the corresponding parameters for the exponential kernel. Note the different vertical scales.

To get a sense of the behavior of Eq (6), we numerically solve the equations on a domain [0, 80] with a spatial discretization of dx = 0.2 and *σ*_*e*_ = 1. We evoke a wave by setting *s*_*e*_(*x*, 0) = 0.2 for *x* ∈ [0, 4]. Fig 9 shows the results of these simulations for the exponential (top row) and Gaussian (middle row) kernels. The figure also shows plots at *x* = 40 for *s*_*e*_, *s*_*i*_, *z* and *s*_*e*_(60, *t*) in the bottom row for the exponential kernel. The wave is faster for the exponential kernel since the spread of excitation decays slower than for the Gaussian. The left most column is the default set of parameters, *g*_*ei*_ = 2, *g*_*ad*_ = 0.25, *σ*_*i*_ = 0.5, so that the spread of inhibition is half that of the excitation. With either of *g*_*ei*_, *g*_*ad*_ removed, the excitation increases in magnitude and persists for a longer time. This can be seen clearer in the bottom plots. The velocity of the wave also increases as can be seen from the smaller slope. Increasing the spatial extent of inhibition to match that of the excitation (*σ*_*i*_ = 1) has the effect of slowing the wave down. In addition, one can see a small oscillation near the onset of the wave in the exponential kernel case. We will spend some time exploring this oscillatory instability later in the paper.

#### Continuation and reduction to a BVP

We would like to more systematically study the behavior of traveling wave solutions to Eq (6) as we vary parameters. Thus, we convert to traveling wave coordinates, *ξ* = *x* + *ct*, so that Eq (6) can be written as:

$$\begin{array}{ccc} c\frac{ds_{e}\left(\xi \right)}{d\xi } & = & -s_{e}/\tau_{e}+F\left(g_{ee}\gamma_{e}S_{e}\left(\xi \right)-g_{ei}\gamma_{i}S_{i}\left(\xi \right)-g_{ad}\gamma_{z}z\left(\xi \right),d_{e}\right)\\ c\frac{ds_{i}\left(\xi \right)}{d\xi } & = & -s_{i}/\tau_{i}+F\left(g_{ie}\gamma_{e}S_{e}\left(\xi \right)-g_{ii}\gamma_{i}S_{i}\left(\xi \right),d_{i}\right)\\ c\frac{dz(\xi )}{d\xi } & = & -z/\tau_{e}+F\left(g_{ee}\gamma_{e}S_{e}\left(\xi \right)-g_{ei}\gamma_{i}S_{i}\left(\xi \right)-g_{ad}\gamma_{z}z\left(\xi \right),d_{e}\right) \end{array}$$ (7)

Let $\left(\bar{s_{e}},\bar{s_{i}},\bar{z}\right)$ be the equilibrium value of the medium at rest. The solutions, (*s*_*e*_(*ξ*), *s*_*i*_(*ξ*), *z*(*ξ*)) must approach this equilibrium as *ξ* → ±∞. Unfortunately, this is an integro-differential equation and while there are some recent numerical tools for solving this type of equation [37], they are not yet well-developed and generally applicable. However, if we choose the exponential kernel, then we can readily invert the convolution to obtain a second order ODE and thus reduce Eq (7) to an ODE. Specifically, if

$$U\left(x\right)=\frac{1}{2\sigma }\int_{-\infty }^{\infty }e^{-|x-y|/\sigma }u\left(y\right)\;dy$$

then

$$\sigma^{2}U_{xx}=U\left(x\right)-u\left(x\right).$$

Thus we can write *S*_*e*_(*ξ*), *S*_*i*_(*ξ*) as a pair of ODEs and Eq (7) becomes:

$$\begin{array}{ccc} c\frac{ds_{e}\left(\xi \right)}{d\xi } & = & -s_{e}/\tau_{e}+F\left(g_{ee}\gamma_{e}S_{e}\left(\xi \right)-g_{ei}\gamma_{i}S_{i}\left(\xi \right)-g_{ad}\gamma_{z}z\left(\xi \right),d_{e}\right)\\ c\frac{ds_{i}\left(\xi \right)}{d\xi } & = & -s_{i}/\tau_{i}+F\left(g_{ie}\gamma_{e}S_{e}\left(\xi \right)-g_{ii}\gamma_{i}S_{i}\left(\xi \right),d_{i}\right)\\ c\frac{dz(\xi )}{d\xi } & = & -z/\tau_{e}+F\left(g_{ee}\gamma_{e}S_{e}\left(\xi \right)-g_{ei}\gamma_{i}S_{i}\left(\xi \right)-g_{ad}\gamma_{z}z\left(\xi \right),d_{e}\right)\\ \sigma_{e}^{2}\frac{d^{2}S_{e}}{d\xi^{2}} & = & S_{e}-s_{e}\\ \sigma_{i}^{2}\frac{d^{2}S_{i}}{d\xi^{2}} & = & S_{i}-s_{i}. \end{array}$$ (8)

This is a 7-dimensional ODE for which we wish to find a homoclinic orbit that corresponds to the traveling pulse solution. Writing the *S*_*e*_, *S*_*i*_ second order equations as a first order system, the equilibrium for $P=(s_{e},s_{i},z,S_{e},{\dot{S}}_{e},S_{i},{\dot{S}}_{i})$ is $\bar{P}=\left({\bar{s}}_{e},{\bar{s}}_{i},\bar{z},{\bar{s}}_{e},0,{\bar{s}}_{i},0\right)$. Linearizing around $\bar{P}$, we find that there is a two-dimensional unstable manifold and a five-dimensional stable manifold. We use XPPAUT and AUTO to find the homoclinic orbit. More details on how we obtain a starting guess for AUTO are described in the methods.

**Fig 10:**
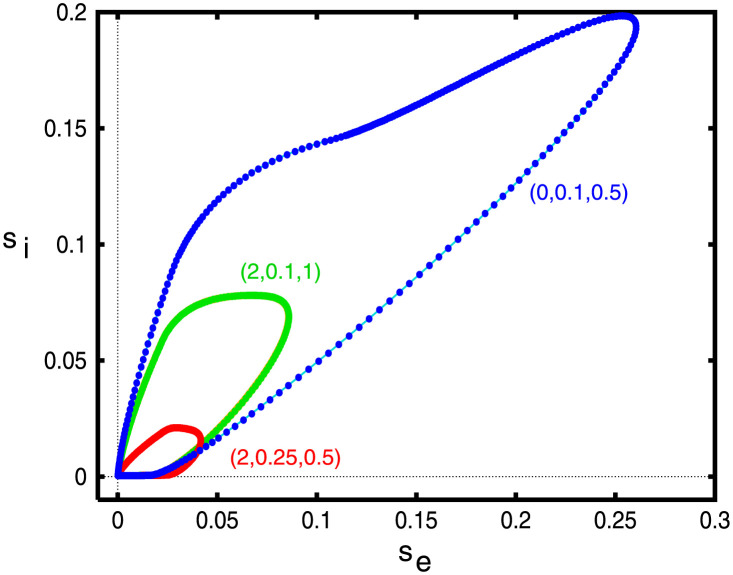
Comparison between solving Eq (6) (thin lines) and the homoclinic computation of Eq (8) (filled circles). Numbers in parentheses correspond to the values of (*g*_*ei*_, *g*_*ad*_, *σ*_*i*_).

We start by verifying that solutions to Eq (8) are identical to the traveling waves obtained by simulation Eq (6). Fig 10 shows three examples with different values of (*g*_*ei*_, *g*_*ad*_, *σ*_*i*_). The plots essentially overlap so that we can be confident that the ODE approach provides the same results as integrating the full system of equations. Importantly, the ODE methods provide a tool that can determine the existence of these waves but it does not let us assess stability. We will explore the stability issue by solving Eq (6) forward in time starting at a known wave solution.

#### Dependence of the wave on parameters

In the next three figures, we hold two of the three parameters, (*g*_*ei*_, *g*_*ad*_, *σ*_*i*_) at fixed values and examine how the velocity varies as a function of the third parameter. We will also look at projections of solutions in the (*s*_*e*_, *s*_*i*_)− and (*s*_*e*_, *z*)− phaseplanes. Later when we study the piece-wise constant version of the model (with smooth nonlinearities replaced by step functions), we will also compute the width and velocity of the pulses and their stability.

**Fig 11:**
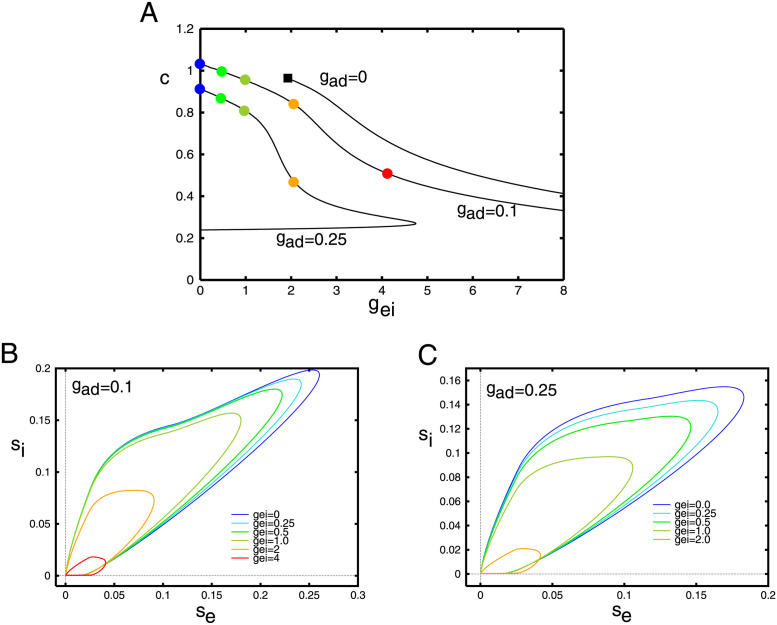
Behavior of Eq (8) as *g*_*ei*_ varies. (A) Velocity of the wave as a function of the strength of the inhibition onto the excitatory cells, *g*_*ei*_ for three different levels of adaptation. Here *σ*_*i*_ = 0.5 is fixed throughout. Colored circles correspond to the parameter values used in the projections onto the (*s*_*e*_, *s*_*i*_)−phaseplanes in panels B,C. Small filled black square shows termination of the pulse to a front (see text).

Fig 11A shows the behavior of the traveling pulse as *g*_*ei*_ changes with *σ*_*i*_ = 0.5 and three values of *g*_*ad*_. At the *g*_*ad*_ = 0.25, the wave exists only if *g*_*ei*_ is smaller than about *g*_*ei*_ = *g*_*SN*_ ≈ 5 where there appears to be a saddle-node bifurcation. For *g*_*ei*_ < *g*_*SN*_, there are two branches with a fast speed and a slow speed. Based on simulations of Eq (6), we believe the fast waves are stable and the slow waves are unstable. (*s*_*e*_, *s*_*i*_) projections of solutions are shown panels (B,C). The amplitudes of both *s*_*e*_, *s*_*i*_ increase as *g*_*ei*_ decreases while the speed of the wave increases. For *g*_*ad*_ = 0.1, we do not find the saddle-node point, at least for the range of *g*_*ei*_ that we varied. For *g*_*ad*_ = 0, the wave seems to stop at about *g*_*ei*_ = 2.0. As we mentioned in the section about local kinetics, at low values of *g*_*ad*_, *g*_*ei*_, the space-clamped system becomes bistable. We will explore this parameter region in the next section.

**Fig 12:**
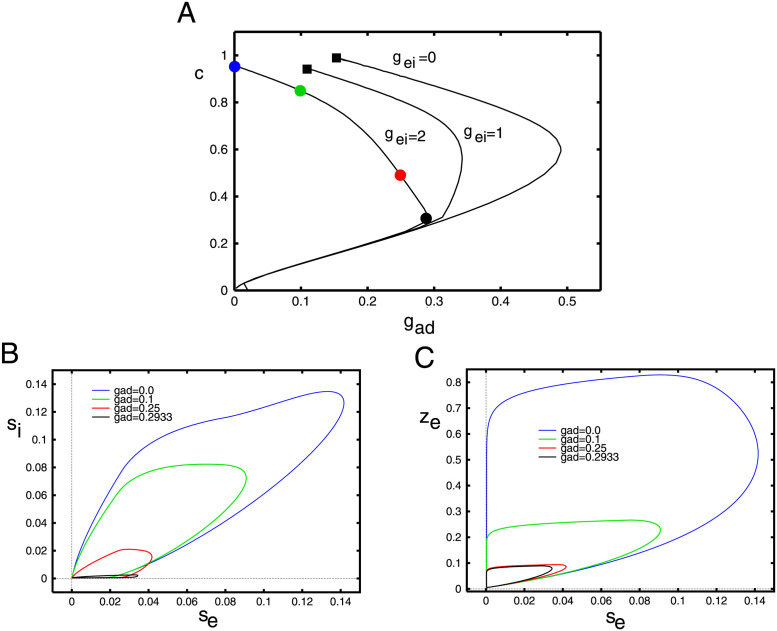
Behavior of Eq (8) as *g*_*ad*_ varies. (A) Velocity of the wave as a function of the adaptation, *g*_*ad*_ for three different values of *g*_*ei*_. Here *σ*_*i*_ = 0.5 is fixed throughout. Colored circles correspond to the parameter values used in the projections onto the (*s*_*e*_, *s*_*i*_)− and (*s*_*e*_, *z*)− phaseplanes in panels B,C. Small filled black squares as in Fig 11.

Fig 12 shows a similar plot where we fix *g*_*ei*_, *σ*_*i*_ and vary *g*_*ad*_, the adaptation. The main difference is that adaptation has a much stronger effect, namely because of the fact that it decays much slower. A small increment in the firing rate is amplified by the adaptation. This can be seen by comparing the vertical axes in panels B,C; inhibition is considerably smaller than adaptation. Additionally, since *E*_*K*_ < *I*_*syn*_, the driving force of adaptation is also larger than that of inhibition. Because of the approximation we made for the adaptation, it is treated just like slow inhibition in the mean-field model. For all values of *g*_*ei*_, there are values of *g*_*ad*_ such that there is a saddle node bifurcation and the wave ceases to exist. Based on the simulations of Eq (6), we believe that the upper (“fast”) branch of solutions is the stable branch of solutions.

In both Figs 11 and 12, we see that when both *g*_*ei*_ and *g*_*ad*_ are small, the pulse ceases to exist (small filled black squares). Unlike the saddle-node bifurcations which mark the termination of any waves, here the pulse wave becomes a *front wave*. In the case of fronts, the local behavior (c.f. Fig 8) is bistable. That is there is a stable low firing state and a stable high firing state. The wave connects the low state to the high state so that eventually the entire network is firing at a high rate. The transition from pulses to waves can be complex and has been well-studied in the reaction-diffusion literature [38].

**Fig 13:**
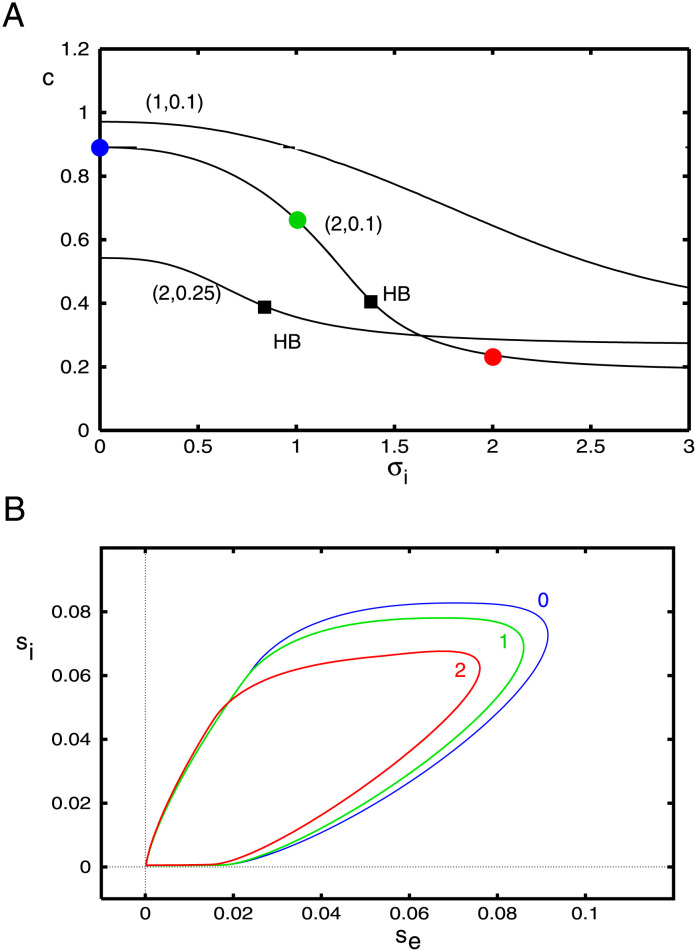
Behavior of Eq (8) as *σ*_*i*_ varies. (A) Velocity of the wave as a function of the spatial decay of inhibition, *σ*_*i*_ for three different pairs (*g*_*ei*_, *g*_*ad*_). Filled circles correspond to the parameter values used in the projections onto the (*s*_*e*_, *s*_*i*_)− phaseplane in panel B. Squares mark apparent Hopf bifurcations of the waves.

Fig 13 shows the wave behavior as a function of the spatial spread of inhibitions for three different pairs of (*g*_*ei*_, *g*_*ad*_). As would be intuitively expected, in all cases, the velocity decreases with the spread of inhibition. The wave appears to exist for *σ*_*i*_ even three times greater than *σ*_*e*_. The shape of the wave in the (*s*_*e*_, *s*_*i*_)− phaseplane shows just a small effect of the inhibitory spread. However, we find that *σ*_*i*_ induces some interesting effects on the stability of the traveling pulse. In the next part of the paper, we will simulate the waves via Eq (6) to assess the stability of the solutions shown in this part of the paper. We find that for *g*_*ei*_ large enough, the traveling wave loses stability as *σ*_*i*_ increases, leading to an apparent Hopf bifurcation. The critical values of *σ*_*i*_ are depicted by the filled black squares in panel A.

#### Instabilities of the wave

**Fig 14:**
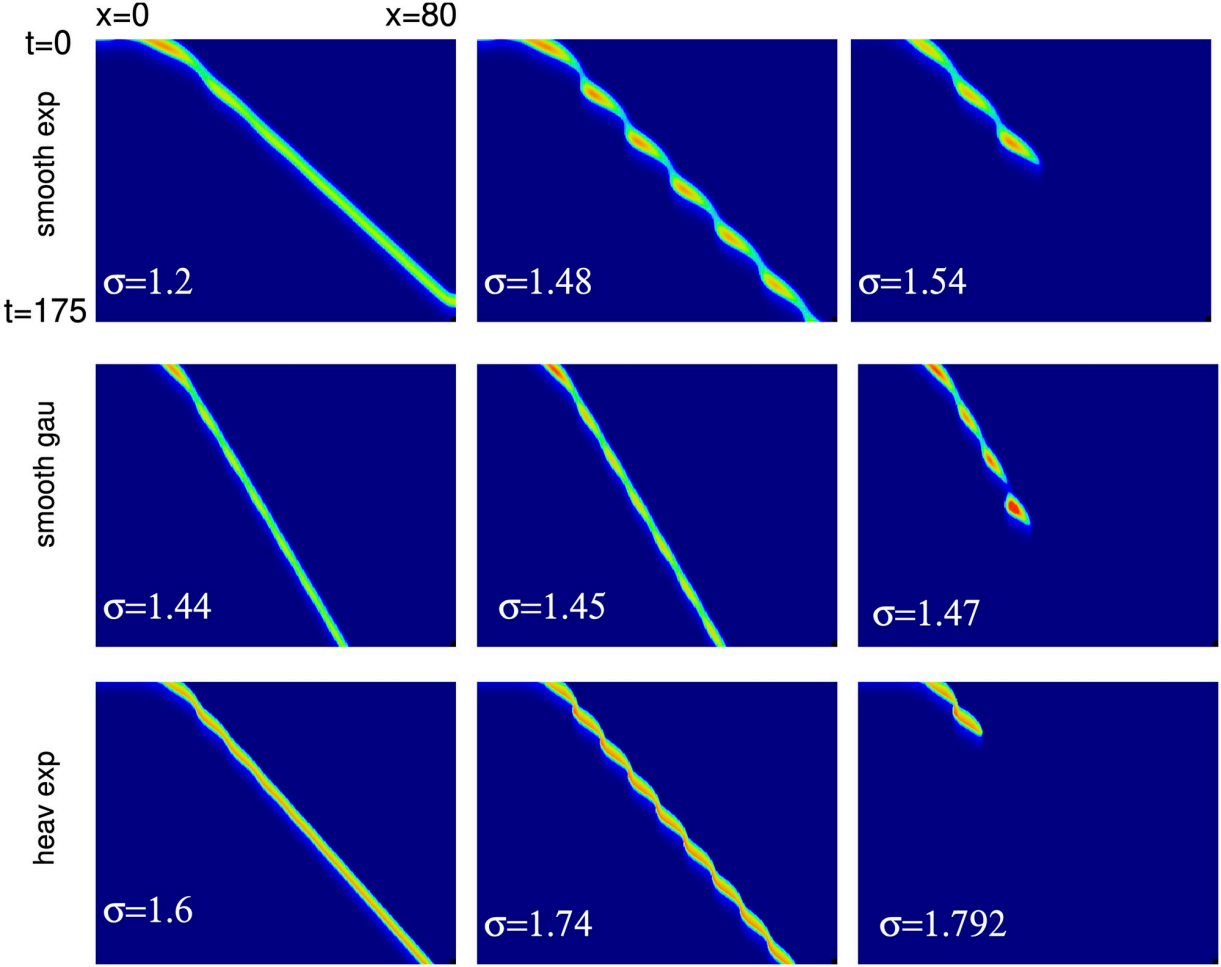
Simulations of the traveling pulse for Eq (6) with (*g*_*ei*_, *g*_*ad*_) = (2.0, 0.1) for different values of *σ*_*i*_. Top row: smooth firing rate function (2) and exponential kernel; middle row: smooth firing rate, Gaussian kernel; last row: Heaviside firing rate function, exponential kernel.

We have used the solutions obtained by our shooting methods as initial data in Eq (6) and then solved the resulting equations forward in time to test stability. In the cases where *g*_*ei*_ or *g*_*ad*_ vary, but *σ*_*i*_ = 0.5 is fixed, we have found that the solutions on the upper velocity curve appear to be stable. For example, when *g*_*ad*_ = 0.1, we have increased *g*_*ei*_ to 8 and find that the wave persists. However, this does not seem to be the case when we increase *σ*_*i*_, the spread of inhibition. Fig 13 shows that the wave *exists* up to at least *σ*_*i*_ = 3. On the other hand, Fig 14 (top) shows the result of starting near the exact traveling wave for three values of *σ*_*i*_. For *σ*_*i*_ = 1.2 (recall that *σ*_*e*_ = 1) one can start to see a bit of oscillation in the wave propagation, but it damps out and all eventually becomes the regular wave. When *σ*_*i*_ = 1.48 the wave propagates but there is a spatiotemporal modulation of the wave. It appears that there is a Hopf bifurcation to an oscillatory modulated wave as the spread of inhibition increases. Destabilization of traveling pulses has also been reported in other work [23, 39] and appears to be through a similar mechanism. The modulated wave finally seems to break down once *σ*_*i*_ gets too large (here, around *σ*_*i*_ ≈ 1.54). This behavior is not confined to the exponential kernel, nor does it require a smooth firing rate function as can be seen in the middle and lower rows respectively.

#### Low adaptation

**Fig 15:**
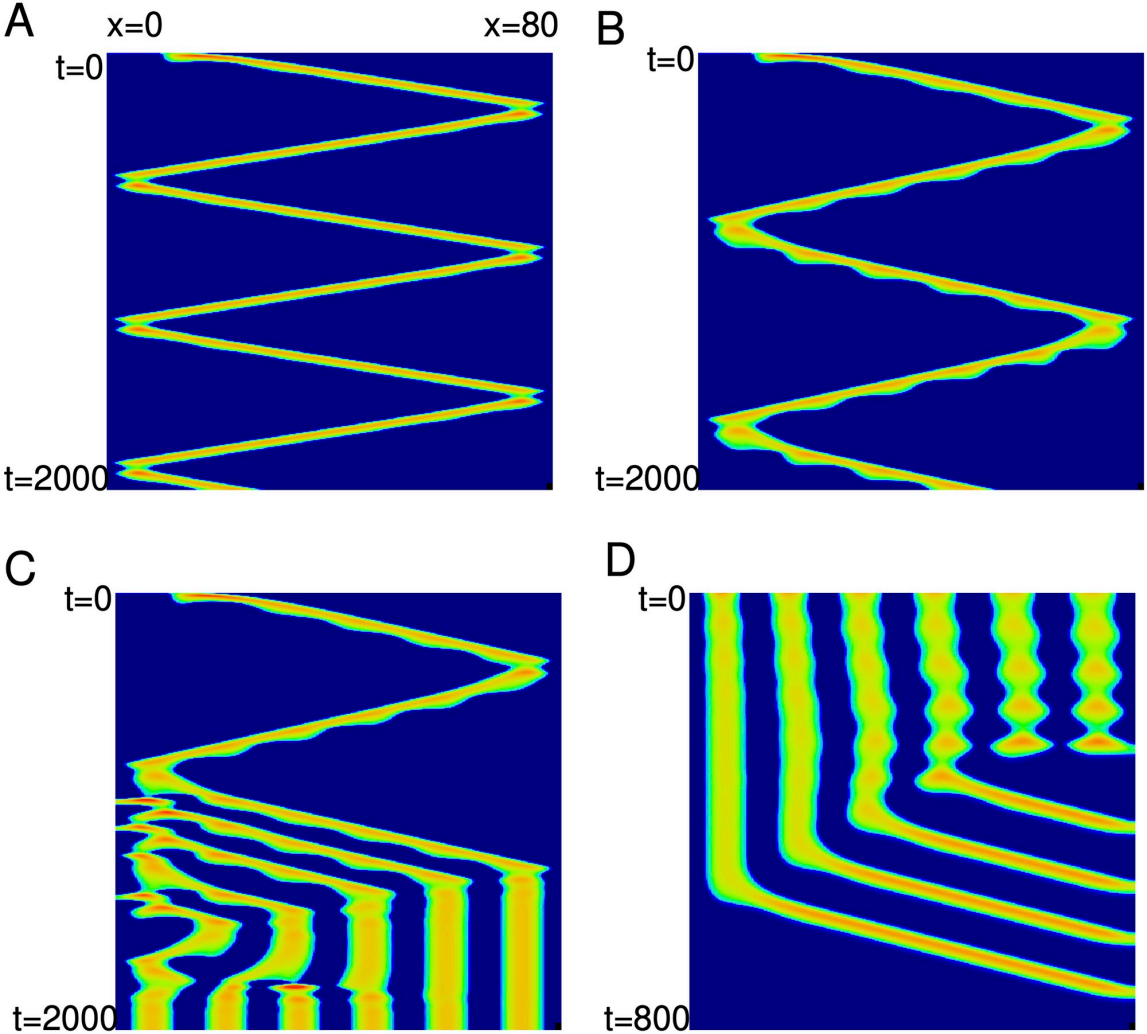
Complex dynamics without adaptation, *g*_*ad*_ = 0. (A) *σ*_*i*_ = 1.6; (B) *σ*_*i*_ = 1.636; (C) *σ*_*i*_ = 1.64; (D) *σ*_*i*_ = 1.51.

Additional bifurcations and pattern formation as well as multistability can be found in Eq (6) when the adaptation is small or turned off. Fig 15 shows some examples of the dynamics of the solitary traveling wave when *g*_*ad*_ = 0 and *σ*_*i*_ increases. (We remark that the same transitions are seen for *g*_*ad*_ nonzero but sufficiently small.) In Panel A, the waves appears to “bounce” off the boundaries forming repeated zigzag waves. As *σ*_*i*_ increases these waves develop periodic modulations (panel B) such that further increases in *σ*_*i*_ break up into regular stripes (panel C). These stripes persist for larger *σ*_*i*_. They are large amplitude and, since the rest state remains asymptotically stable, they do not directly emerge as Turing patterns. These stripes coexist with the zigzag waves and the solitary traveling waves for a range of *σ*_*i*_. In panel D, we start at the striped initial conditions but lower *σ*_*i*_ to 1.51 and observe the stripes break up into a series of traveling pulses.

#### Heaviside approximation

**Fig 16:**
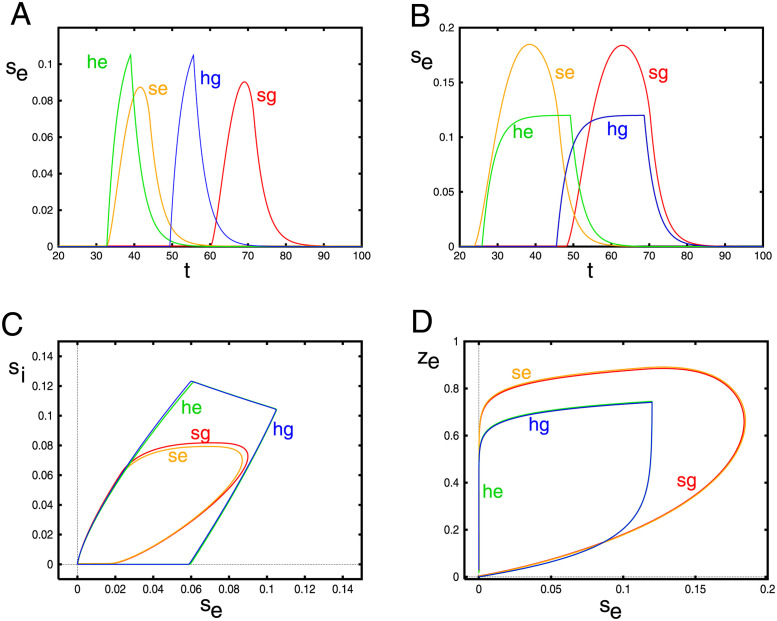
Comparison between the smooth model (s) and the Heaviside (h) models for the exponential (e) and Gaussian (g) kernels. In (A, C),(*g*_*ei*_, *g*_*ad*_, *σ*_*i*_) = (2, 0.1, 1); in (B,D) *g*_*ei*_ = 0, *g*_*ad*_ = 0.25 Time series plots show *s*_*e*_(40, *t*) and phaseplanes show *s*_*i*_(40, *t*) and *z*(40, *t*) on the *y*-axes.

In order to get some more insight into the behavior of Eq (6) as well as explore the instabilities, we will approximate the nonlinearity (2) by a step function and then rescale to put it into a simpler form that is suitable for analysis. The first step is to choose parameters, *a*_1_, *a*_2_, so that if we replace the smooth *F*(*G*, *d*) with *a*_1_*H*(*G* − *a*_2_), then, quantities such as the wavespeed and amplitudes are similar at some nominal parameter value. We pick (*g*_*ei*_, *g*_*ad*_, *σ*_*i*_) = (2, 0.1, 1), we choose *a*_1_ = 0.04, *a*_2_ = 0.12 so that the velocity of the waves matches that of the exponential kernel with smooth nonlinearity. Fig 16 shows the comparison of the times series for *s*_*e*_(40, *t*) and phaseplane projections for the smooth and Heaviside function nonlinearities as and exponential and Gaussian kernels. Panels A,B show that while the heights do not match well (the step function saturates), the widths are roughly the same as is the velocity. For the Gaussion kernel, with our choice of parameters, the step function wave is much faster than the smooth wave. In panel B, we remove all the inhibition and increase *g*_*ad*_ to 0.25. Here both the velocity and width still match and the results for the Gaussian kernel are more similar. In panels (C,D), the projections into the phaseplanes are shown. The plots are all qualitatively similar.

#### Analysis of the Heaviside model

As a first step, we replace *s*_*e*_, *s*_*i*_, *z* by $a_{1}\tau_{e}{\hat{s}}_{e}$, $a_{1}\tau_{i}{\hat{s}}_{i}$, and $a_{1}\tau_{z}\hat{z}$ respectively and then drop the hats over the variables to obtain a “normalized” step function model:

$$\begin{array}{ccc} \tau_{e}\frac{\partial s_{e}\left(x,t\right)}{\partial t} & = & -s_{e}\left(x,t\right)+H\left(g_{ee}\kappa_{e}S_{e}\left(x,t\right)-g_{ei}\kappa_{i}S_{i}\left(x,t\right)-g_{ad}\kappa_{z}S_{z}\left(x,t\right)-a_{2}\right)\\ \tau_{i}\frac{\partial s_{i}\left(x,t\right)}{\partial t} & = & -s_{i}\left(x,t\right)+H\left(g_{ie}\kappa_{e}S_{e}\left(x,t\right)-g_{ii}\kappa_{i}S_{i}\left(x,t\right)-a_{2}\right)\\ \tau_{z}\frac{\partial z(x,t)}{\partial t} & = & -z\left(x,t\right)+H(g_{ee}\kappa_{e}S_{e}\left(x,t\right)-g_{ei}\kappa_{i}S_{i}\left(x,t\right)-g_{ad}\kappa_{z}S_{z}\left(x,t\right)-a_{2}) \end{array}$$

where *κ*_*β*_ = *a*_1_*τ*_*β*_*γ*_*β*_. We replace *z*(*x*, *t*) in the adaptation by

$$S_{z}\left(x,t\right)=\left(1/\sigma_{z}\right)\int_{R}W\left(\left(x-y\right)/\sigma_{z}\right)z\left(y,t\right),$$

where we let *σ*_*z*_ be very small. As we will see below, the reason for this is to avoid some technical difficulties when analyzing the stability. Now *s*_*e*_, *s*_*i*_, *z* all take values between 0 and 1. For our parameter choices *κ*_*e*_ = 0.92, *κ*_*i*_ = 0.37333, and *κ*_*z*_ = 7.333333.

To study the existence and stability of traveling waves, we replace *x* by the moving variable, *ξ* = *ct* + *x*. Traveling waves are then just solutions independent of *t*. In this coordinate system, we obtain

$$\begin{array}{ccc} \tau_{e}\frac{\partial s_{e}\left(\xi ,t\right)}{\partial t}+c\tau_{e}\frac{\partial s_{e}\left(\xi ,t\right)}{\partial \xi } & = & -s_{e}\left(\xi ,t\right)+H\left(g_{ee}\kappa_{e}S_{e}\left(\xi ,t\right)-g_{ei}\kappa_{i}S_{i}\left(\xi ,t\right)-g_{ad}\kappa_{z}S_{z}\left(\xi ,t\right)-a_{2}\right)\\ \tau_{i}\frac{\partial s_{i}\left(\xi ,t\right)}{\partial t}+c\tau_{i}\frac{\partial s_{i}\left(\xi ,t\right)}{\partial \xi } & = & -s_{i}\left(\xi ,t\right)+H\left(g_{ie}\kappa_{e}S_{e}\left(\xi ,t\right)-g_{ii}\kappa_{i}S_{i}\left(\xi ,t\right)-a_{2}\right)\\ \tau_{z}\frac{\partial z(\xi ,t)}{\partial t}+c\tau_{z}\frac{\partial z(\xi ,t)}{\partial \xi } & = & -z\left(\xi ,t\right)+H(g_{ee}\kappa_{e}S_{e}\left(\xi ,t\right)-g_{ei}\kappa_{i}S_{i}\left(\xi ,t\right)-g_{ad}\kappa_{z}S_{z}\left(\xi ,t\right)-a_{2}). \end{array}$$ (9)

Steady states can be written as *s*_*e*_(*ξ*, *t*) = *u*_*e*_(*ξ*) etc where each *u*_*β*_(*ξ*) must vanish as *ξ* → ±∞. Let

$$I_{\beta }\left(\xi \right)=g_{\beta e}\kappa_{e}R_{e}\left(\xi \right)-g_{\beta i}\kappa_{i}R_{i}\left(\xi \right)-g_{ad}\kappa_{z}R_{z}\left(\xi \right)-a_{2}$$

for *β* ∈ {*e*, *i*} and *R*_*e*,*i*,*z*_(*ξ*) are the convolutions of *u*_*e*,*i*,*z*_(*ξ*) with their respective kernels. (Note that there is no adaptation for the inhibitory cells.) The step function is 0 (1) if *I*_*β*_(*ξ*) is negative (resp., positive). Let *I*_*e*_(*ξ*) be positive for 0 < *ξ* < *a*, and *I*_*i*_(*ξ*) be positive for *b* < *ξ* < *d*. Our first goal is the solve for (*c*, *a*, *b*, *d*) whose values characterize the wave. (Note that since the waves are translation invariant, we can shift *ξ* so that *u*_*e*_(*ξ*) “turns on” at *ξ* = 0.) By continuity, we must have *I*_*e*_(0) = *I*_*e*_(*a*) = 0 and *I*_*i*_(*b*) = *I*_*i*_(*d*) = 0 along with the requirement that the derivatives of *I*_*e*,*i*_(*ξ*) with respect to *ξ* be nonzero at these crossings. Given (*c*, *a*, *b*, *d*) we can solve for *u*_*e*,*i*,*z*_(*ξ*) as they solve

$$c\tau_{\beta }\frac{du_{\beta }}{d\xi }=-u_{\beta }\left(\xi \right)+H\left(I_{\beta }\left(\xi \right)\right)$$

(where *I*_*z*_(*ξ*) = *I*_*e*_(*ξ*)). For example:

$$u_{i}\left(\xi \right)=\{\begin{array}{cc} 0 & \xi <b\\ 1-\text{exp}(-\left(\xi -b\right)/\left(c\tau_{i}\right)) & b<\xi <d\\ \left(1-\text{exp}\left(-\left(d-b\right)/\left(c\tau_{i}\right)\right)\right)\;\text{exp}\left(-\left(\xi -d\right)/\left(c\tau_{i}\right)\right) & d<\xi \end{array},$$

with similar expressions for *u*_*e*_(*ξ*) and *u*_*z*_(*ξ*). Given these functions, we can then evaluate the convolutions, *R*_*e*,*i*,*z*_(*ξ*) and thus obtain *I*_*e*_(*ξ*), *I*_*i*_(*ξ*) in terms of (*ξ*, *c*, *a*, *b*, *d*). Setting them to zero at the appropriate values of *ξ* gives us four equations in four unknowns:

$$\begin{array}{ccc} I_{e}\left(0\right)\equiv F_{1}\left(c,a,b,d\right) & = & 0\\ I_{e}\left(a\right)\equiv F_{2}\left(c,a,b,d\right) & = & 0\\ I_{i}\left(b\right)\equiv F_{3}\left(c,a,b,d\right) & = & 0\\ I_{i}\left(d\right)\equiv F_{4}\left(c,a,b,d\right) & = & 0. \end{array}$$

We get good guesses for (*c*, *a*, *b*, *d*) using the simulation of Eq (6) (with the step function nonlinearity) and then using a root finder. The functions *F*_*j*_ are complicated but easily obtained from the integrals using a symbolic algebra package.

**Fig 17:**
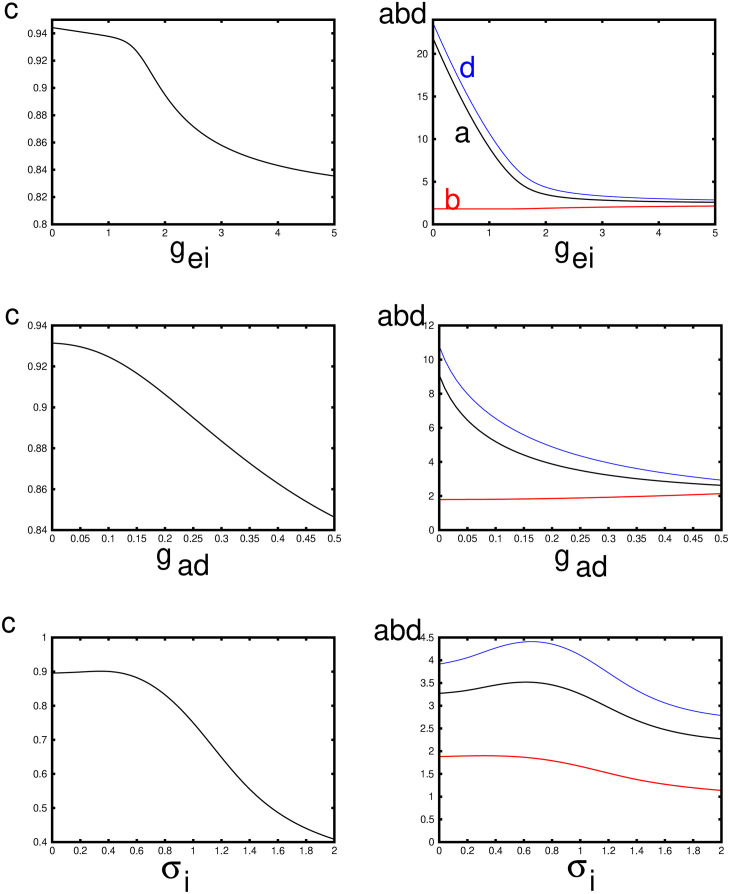
Properties of the traveling wave for the Heaviside firing rate function and the exponential kernel as parameters vary. The baseline values are (*g*_*ei*_, *g*_*ad*_, *σ*_*i*_) = (2, 0.25, 0.5). Here *c* is the wave speed, *a* is the width of the suprathreshold input to the excitatory population, *b* is the onset of the inhibition, and *d* is the offset. *b* − *d* is the width of the suprathreshold input to the inhibitory population.

Fig 17 shows how various parameters related to the shape of the pulse vary as the three main parameters, *g*_*ei*_, *g*_*ad*_, *σ*_*i*_ vary around their nominal values. In particular, the speed, *c* can be compared to the behavior of the smooth nonlinearity, Figs 11–13. The speed decreases with any increases in these three parameters. Recall that *a* is the value of *ξ* where the input into the excitatory population falls back below threshold so that *a* is a surrogate for the width of the excitatory pulse. In fact, it is the distance from to time that excitation starts to the point that it reaches its peak. As intuitively expected, the width increases with reduced inhibition and adaptation. but has a nonmonotonic behavior with respect to *σ*_*i*_. For *ξ* ∈ (*b*, *d*), the input to the inhibition is above threshold; as with excitation, *b* marks the onset of firing for the inhibitory population and *d* marks its peak.

#### Stability of the wave

The big advantage of the Heaviside function approach is that it is possible to determine the stability of the waves. In order to do this, we need to formally linearize Eq (9) about the traveling wave solutions, (*u*_*e*_(*ξ*), *u*_*i*_(*ξ*), *u*_*z*_(*ξ*)). Let *s*_*e*_(*ξ*, *t*) = *u*_*e*_(*ξ*) + exp(λ*t*)*v*_*e*_(*ξ*) with similar expressions for *s*_*i*_(*ξ*, *t*), *z*(*ξ*, *t*) where λ and *v*_*β*_(*ξ*) are to be obtained. The wave will be unstable if ℜλ > 0. Plugging this into Eq (9) and retaining the linear terms, we obtain the following equations:

$$\begin{array}{ccc} \lambda \tau_{e}v_{e}\left(\xi \right)+c\tau_{e}\frac{dv_{e}}{d\xi } & = & -v_{e}\left(\xi \right)+\delta \left(I_{e}\left(\xi \right)\right)\Gamma_{e}\left(\xi \right)\\ \lambda \tau_{i}v_{i}\left(\xi \right)+c\tau_{i}\frac{dv_{i}}{d\xi } & = & -v_{i}\left(\xi \right)+\delta \left(I_{i}\left(\xi \right)\right)\Gamma_{i}\left(\xi \right)\\ \lambda \tau_{z}v_{z}\left(\xi \right)+c\tau_{z}\frac{dv_{z}}{d\xi } & = & -v_{z}\left(\xi \right)+\delta \left(I_{e}\left(\xi \right)\right)\Gamma_{e}\left(\xi \right)\\ \Gamma_{e}\left(\xi \right) & = & \left[g_{ee}\kappa_{e}Q_{e}\left(\xi \right)-g_{ei}\kappa_{i}Q_{i}\left(\xi \right)-g_{ad}\kappa_{z}Q_{z}\left(\xi \right)\right]\\ \Gamma_{i}\left(\xi \right) & = & \left[g_{ie}\kappa_{e}Q_{e}\left(\xi \right)-g_{ii}\kappa_{i}Q_{i}\left(\xi \right)\right] \end{array}$$ (10)

where *Q*_*β*_(*ξ*) are the convolutions of the variables, *v*_*β*_(*ξ*) with their respective kernels and the delta function is interpreted as:

$$\delta \left(I\left(\xi \right)\right)=\frac{\delta (\xi -\xi_{1})}{|I^{\prime }\left(\xi_{1}\right)|}+\frac{\delta (\xi -\xi_{2})}{|I^{\prime }\left(\xi_{2}\right)|}$$

where *ξ*_1.2_ are the values of *ξ* where *I*(*ξ*) crosses 0. For excitation and adaptation, *ξ*_1_ = 0, *ξ*_2_ = *a* and for inhibition, *ξ*_1_ = *b*, *ξ*_2_ = *d*. Note that our “transversility” requirement implies that the derivatives of *I*_*β*_(*ξ*) at these points are nonzero. Before turning to the analysis of this equation, we first explain why we have convolved the adaptation with a sharp kernel. In each equation in the system (10) the delta functions are multiplied by Γ_*β*_(*ξ*). Consider the equation for *v*_*z*_(*ξ*). At *ξ* = 0 *v*_*z*_(*ξ*) must jump (since its derivative is a delta function). However, if we replaced *Q*_*z*_(*ξ*) with *v*_*z*_(*ξ*), then Γ_*e*_(*ξ*) would have a discontinuity at *ξ* = 0, so it is not clear how to evaluate the jump in *v*_*z*_(*ξ*) at *ξ* = 0. For this reason, we convolve *v*_*z*_(*ξ*) with a narrow kernel to assure that Γ_*e*_(*ξ*) is continuous at *ξ* = 0 (and *ξ* = *a*) and avoid this technical difficulty.

Consider *v*_*e*_(*ξ*) first. (The other two follow similarly.) We can write:

$$\frac{dv_{e}}{d\xi }=-v_{e}/\zeta +A_{0}\delta \left(\xi \right)+A_{a}\delta \left(\xi -a\right)$$

where *A*_0,*a*_ are the jumps for *v*_*e*_(*ξ*) and *ζ*_*e*_ = *cτ*_*e*_/(λ*τ*_*e*_ + 1). The solution to this equation is:

$$v_{e}\left(\xi \right)=A_{0}\;\text{exp}\left(-\xi /\zeta_{e}\right)H\left(\xi \right)+A_{a}\;\text{exp}\left(-\left(\xi -a\right)/\zeta_{e}\right)H\left(\xi -a\right).$$

We similarly obtain:

$$\begin{array}{ccc} v_{z}\left(\xi \right) & = & A_{0}\;\text{exp}\left(-\xi /\zeta_{z}\right)H\left(\xi \right)+A_{a}\;\text{exp}\left(-\left(\xi -a\right)/\zeta_{z}\right)H\left(\xi -a\right)\\ v_{i}\left(\xi \right) & = & A_{b}\;\text{exp}\left(-\left(\xi -b\right)/\zeta_{i}\right)H\left(\xi -b\right)+A_{d}\;\text{exp}\left(-\left(\xi -d\right)/\zeta_{i}\right)H\left(\xi -d\right), \end{array}$$

where *ζ*_*z*,*i*_ are defined similarly to *ζ*_*e*_. The size of the jumps, *A*_0,*a*,*b*,*d*_ is found by plugging 0, *a*, *b* of *d* into Γ_*β*_(*ξ*) after dividing by the slopes $|I_{\beta }^{\prime }\left(\xi \right)|$ and *cτ*_*β*_. Let $\vec{A}$ be the column vector (*A*_0_, *A*_*a*_, *A*_*b*_, *A*_*d*_)^*T*^. Then we obtain

$$\vec{A}=\text{M}\left(\lambda \right)\vec{A}$$

where **M**(λ) is a 4 × 4 matrix obtained from evaluating Γ_*β*_. For example, Γ_*e*_(0) depends on convolutions of *v*_*e*,*i*,*z*_ evaluated at *ξ* = 0 and these depend linearly on *A*_0,*a*,*b*,*d*_. The coefficients multiplying the *A*_0,*a*,*b*,*d*_ are then the entries of **M**(λ). We emphasize that each entry in **M** depends on the unknown eigenvalue, λ. The equation for $\vec{A}$ has a nontrivial solution if and only if

E(λ)≡det(M-I)=0.

The complex function, *E*(λ) is called the Evans function [24, 26, 27]. The zeros of this function are the eigenvalues for the linearized system. We set *σ*_*z*_ = 0.001 in all the subsequent calculations; thus, *Q*_*z*_(*ξ*) is quite close to *v*_*z*_(*ξ*) but is continuous in *ξ*. We grid an area in the complex plane around λ = 0, and use MATLAB to compute *E*(λ). We then plot the zero contours of the real and imaginary parts of *E*(λ). Intersections of these contours are the eigenvalues. For each choice of parameters, we need to determine *c*, *a*, *b*, *d* and then compute the relevant integrals at the values of *ξ* ∈ {0, *a*, *b*, *d*}.

**Fig 18:**
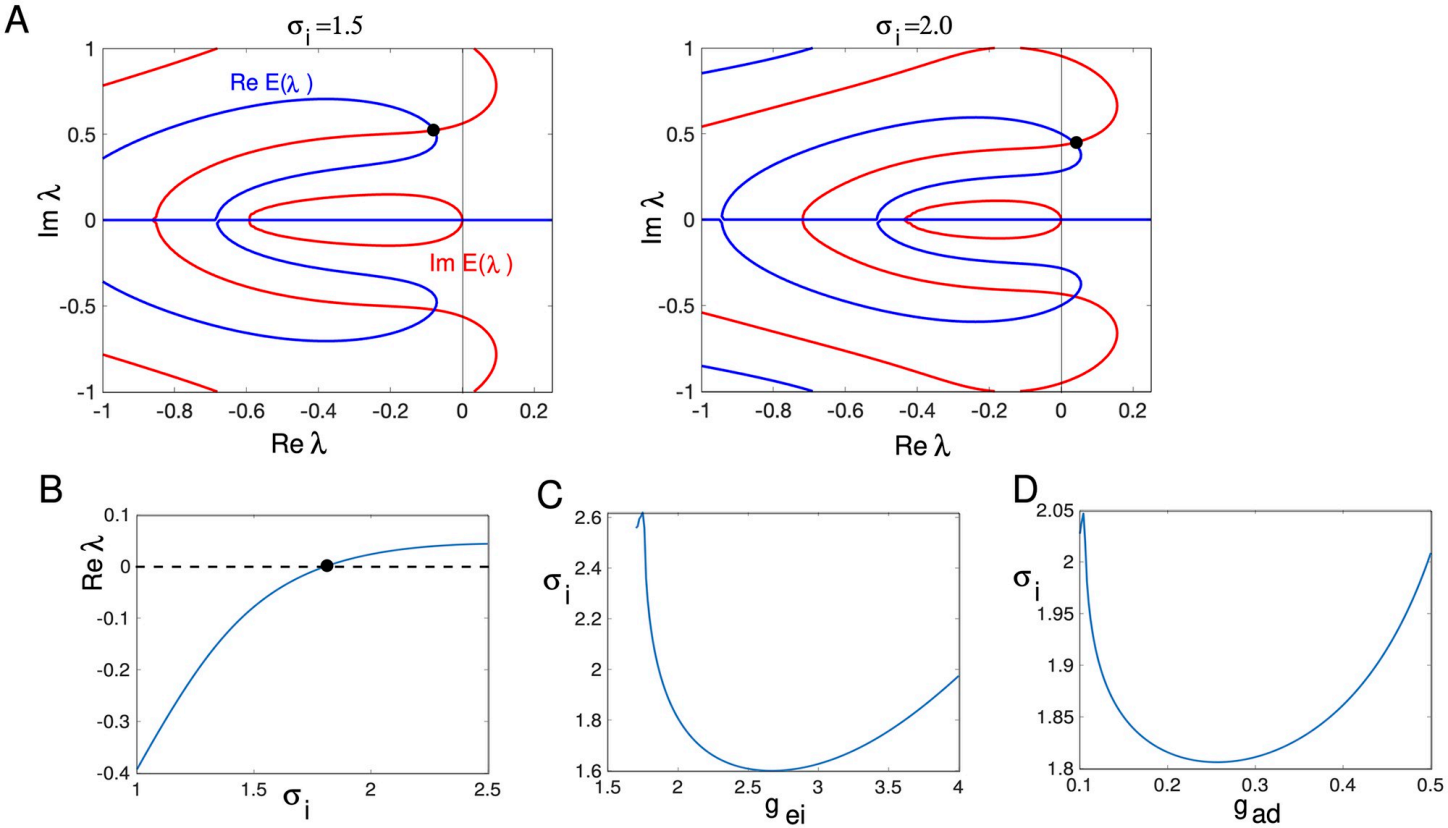
Stability of the traveling pulse. (A) The contours of the real and imaginary parts of the Evans function *E*(λ) whose intersections are the eigenvalues. *g*_*ei*_ = 2.0, *g*_*ad*_ = 0.25 and *σ*_*i*_ is indicated in the figure. (B) Real part of the maximal nonzero eigenvalue as *σ*_*i*_ varies. Zero crossing is a Hopf bifurcation. (C,D) Value of *σ*_*i*_ where there is a Hopf bifurcation as *g*_*ei*_ or *g*_*ad*_ vary.

Recall Fig 14 suggests that as *σ*_*i*_ increases, the waves lose stability via a Hopf bifurcation. Thus, we consider the stability of the wave as we vary *σ*_*i*_. We fix *g*_*ei*_ = 2, *g*_*ad*_ = 0.25. Fig 18 shows the results of this stability calculation. The top panel shows the contours of the real and imaginary parts of *E*(λ) as λ is varied in the region [−1, 0.2] + *i*[−1, 1]. The blue (red) curves show the real (imaginary) zero contours. They always intersect at λ = 0 corresponding to the translation invariance of the wave. For *σ*_*i*_ = 1.5, there is a complex eigenvalue with a negative real part that is close to 0 (indicated by the filled circle). When *σ*_*i*_ = 2.0, this intersection has moved over to the right half plane indicating that the wave is unstable. In Panel B, we follow this eigenvalue as we vary *σ*_*i*_ and see that at *σ*_*i*_ ≈ 1.8, the real part of the eigenvalue changes from negative to positive. At this point, the imaginary part is approximately, 0.45, indicating the possibility of a Hopf bifurcation. The numerical simulations we did above indicate that it is supercritical and a periodically modulated wave emerges for a narrow range of values of *σ*_*i*_. In Panels C and D, we find the critical value of *σ*_*i*_ where ℜλ = 0 as we covary *σ*_*i*_ and either *g*_*ei*_ or *g*_*ad*_. In both cases, the dependence of *σ*_*i*_ is nonmonotone. We were not able to make *g*_*ei*_ (or *g*_*ad*_) too small before the calculation failed. In sum, our stability calculations indicate that as the spatial spread of inhibition increases, the traveling pulse loses stability through a complex eigenvalue. For a limited range of *σ*_*i*_ beyond this critical value, there are periodically modulated waves. These waves eventually break up and the pulse can no longer propagate.

## Discussion

In this paper, we have explored the role of inhibition and adaptation on the control and propagation of traveling pulses in cortical networks. Starting with a spiking model, we showed that at high levels of recurrent inhibition, only about 10% of the excitatory neurons fire during a pulse and this is consistent with the small amplitude and somewhat irregular LFP seen in the experiment Fig 2. Lowering the amount of inhibition led to both faster waves and much greater participation of the excitatory neurons. While there remain many questions about the computational role for waves [1], one possibility is that a wave of activity can depolarize neurons sufficiently to prime them for a later stimulus [17]. We demonstrated such an effect in Fig 5 in the spiking network.

To gain better insight into how parameters change the properties of the waves, we derived a mean-field approximation for the spiking model. Using simulation and numerical shooting, we were able to study how the velocity and magnitude of the waves changed as parameters relating to negative feedback were changed. In particular, we found that without enough adaptation, as inhibition is reduced, the pulse wave is lost to a front. Conversely, as the strength or spread of inhibition increases, the pulse wave is lost through two mechanisms. First, it can be lost through a saddle-node bifurcation; as the inhibition increases, no wave exists. This loss of existence occurs at a finite value of velocity, *c* (c.f. Figs 11 and 12). For this reason, we have not been able to get the same dynamic range of velocity as is seen in the experiments, (e.g. Fig 2). The biggest dynamic range that we find is about 5-fold. The recent model by Gonzalez-Ramirez [13] has a similar range in velocities as we do. One possible explanation for the wide range in velocities seen in slice is that there are other sources of inhibition that are not directly dependent on the excitation. This “background inhibition” could act to increase the threshold to firing of the excitatory neurons which could lower the minimal velocity of the traveling waves. The second mechanism through which constant speed traveling waves are lost is through an apparent Hopf bifurcation. This instability leads to periodically modulated waves as seen in Fig 14. In this case, we changed the “footprint” (spatial spread) of the inhibition, and eventually, the wave fails to propagate. In the Heaviside approximation, we show that changing either the strength of adaptation or inhibition can have a similar destabilizing effect, c.f. Fig 18. Other interesting effects that we found in the smooth case are waves that reflect off the boundaries and transition to pattern formation (Fig 15); the exact bifurcations which underly this are not completely resolved.

Our present work is similar to the paper by Gonzalez-Ramirez et al [13] in that both models study waves in networks with excitatory and inhibitory neurons in addition to a slow adaptation that serves to suppress run-away excitation. Their adaptation is linear whereas ours is nonlinear; both forms of adaptation have an large effect at damping the strong excitation. One main difference between our work and theirs is in the effects of inhibition. In their paper, the main source of control of activity is the adaptation while in ours it is the inhibition. Because adaptation is slow, it cannot control the magnitude of the excitation so that the waves that occur in adaptation-dominated networks are much broader and involve almost all of the excitatory neurons. This can be seen in or spiking simulations in Fig 3 (*g*_*ei*_ = 0) and in Fig 4C. In our work the inhibition has a considerable effect on controlling the width of the wave in contrast to [13] (c.f. Fig 7 in their paper). In their model, inhibition increased the wave speed while in ours and in the experimental results in Fig 2, velocity decreased. They found that the inhibition had to be 10 fold slower than the excitation in order to attain propagation; in our model the time scales of excitation and inhibition were similar. One explanation for these different results is that they were primarily interested in seizure-type waves while our focus as been on sensory evoked waves. The underlying assumptions of the two different models are likely to be different. Their approach to varying parameters is also quite different from ours as throughout the paper they seek analytic expressions for the wave. As a consequence, they treat width and velocity as parameters in order to obtain parametric expressions for other parameters such as the strength of inhibition. We numerically compute velocity and width directly by continuation so we can compare the wave shape and velocity to the parameters relevant to inhibition. Finally, we found a number of bifurcations to more complex wave forms (e.g. periodically modulated waves) as we varied the spatial spread and strength of inhibition which provided us with information on how the waves cease to propagate.

In earlier experimental work [10, 40], these authors show that the ability of waves to initiate and propagate depends on the feedback between layers 2/3 and 5 in the cortex. The dense recurrent excitation in layer 5 coupled with the longer range excitatory interactions underly the initiation and propagation of traveling waves in normal and disinhibited cortex. Thus, a natural extension of the present work is to create a two-layer network and explore how strong recurrent connections in one layer (5) interact with weaker but longer-range connections in the other (2/3) to produce propagation.

In conclusion, in this paper we have tried to explore how inhibitory feedback and intrinsic adaptation work together to control the propagation of waves through space. Sensory evoked waves are one possible mechanism for transmitting information in one cortical area or region to another. Recurrent excitation is required to maintain the transmission in a robust manner. However, this strong excitation must be controlled. Our analysis and simulations suggest ways in which inhibition can keep the activity at reasonable levels while at the same time allowing for long distance propagation.

## Materials and methods

### Experimental methods

#### Ethics statement

Sprague-Dawley rats (P14-P35) and C57BL6 mice (P21-P60) of both sexes were used in accordance with protocols (#05-057, #08-055 and #10-014) approved by Georgetown University Animal Care and Use Committee.

#### Cortical slice preparation

Following deep isoflurane anesthesia, animals were rapidly decapitated. The whole brain was subsequently removed and chilled in cold (0°C) sucrose-based cutting artificial cerebrospinal fluid (sACSF) containing (in mM) 252 sucrose; 3 KCl; 2 CaCl_2_; 2 MgSO_4_; 1.25 NaH_2_PO_4_; 26 NaHCO_3_; 10 dextrose and bubbled by 95% O_2_, 5% CO_2_. Cortical slices (300 um thick) were cut in coronal sections from dorsal to ventral brain with a vibratome (Leica, VT1000S). Slices are incubated in ACSF contained (in mM) NaCl, 132; KCl, 3; CaCl_2_, 2; MgSO_4_, 2; NaH_2_PO_4_, 1.25; NaHCO_3_, 26; dextrose 10; and saturated with 95% O_2_, 5% CO_2_ at 26-27°C. The incubation chamber is equipped with a small pump to circulate oxygenated ACSF for 2-5 hours [41]. In well circulated conditions the slices can recover well from the injury of slicing. Fully recovered slices can remain viable for up to 24 hours [41, 42]. Bicuculline was added through the perfusion system for about 5 minutes. The effects persist until it is removed from the bath.

#### Field potential recording (LFP) in slice

Low resistance glass microelectrodes (50-150KΩ tip resistance) were used for LFP recordings. The electrodes were pulled with a Sutter P87 puller (Sutter Instruments) with 6 controlled pulls. Electrodes were filled with ACSF. The recordings were done in a submerged chamber, and slices were perfused on both sides at a high flow rate (10-30 ml/min). The LFP data are amplified 1000x by a custom-made amplifier (0.01-1000Hz) and digitized at 3000 Hz by a 12-bit USB Analog-to-digital converter (National instruments). From each brain slice we can usually record continuously for 3—8 hours.

#### Voltage-sensitive dye recording

The imaging apparatus and methods are described in detail in [43, 44]. Briefly, the slices were stained with ACSF containing 0.005 to 0.02 mg/ml of an oxonol dye, NK3630 [45] for 30 to 60 minutes. A 124-element photodiode array system was used for the imaging. The preparation was trans-illuminated by 705 ± 20 nm light for the imaging. An objective of 5× (0.12 NA, Zeiss) was used to form the image on the diode array. Each photodetector received light from an area of 330 × 330 *μ*m*m*^2^ of the cortical tissue. With trans-illumination, neurons through the entire thickness of the slice(450 *μ*m) contributed equally to the signal. The resting light intensity was about 109 photons/msec per detector and the VSD signal of the oscillation was about 0.01% (peak-to-peak) the resting light intensity. The signal was AC coupled at 0.1 Hz, amplified 200 times, low-pass filtered at 333 Hz, and then digitized at 1,000 frames /sec with a 12 bit accuracy.

### Spiking model

The spiking model consists of a network of *N*_*e*_ = 400 excitatory (E) and *N*_*i*_ = 80 inhibitory (I) quadratic integrate-and-fire neurons which we transform to a network of theta neurons for ease in integration. For simplicity of later reduction, we use “current” synapses rather than conductance-based synapses. Each excitatory neuron, *j* = 0, …, *N*_*e*_ − 1 is positioned at a spatial location *x* ∈ [0, 1) where *x* = *j*/400 and each inhibitory neurons, *j* = 0, …, *N*_*i*_ − 1 is positioned at *x* = *j*/80. Connections between neurons are made probabilistically and fixed:

$$\begin{array}{ccc} W_{E\to E}\left(x,y\right) & = & 0.35\;\text{exp}(-10|x-y|)<\text{rand}\\ W_{E\to I}\left(x,y\right) & = & \text{exp}(-10|x-y|)<\text{rand}\\ W_{I\to E}\left(x,y\right) & = & \text{exp}(-20|x-y|)<\text{rand}\\ W_{I\to I}\left(x,y\right) & = & \text{exp}(-20|x-y|)<\text{rand} \end{array}$$

where rand is a uniform random number in (0, 1). This produces a fairly sparse distance-dependent network. Each E cell receives about 27 excitatory and 8 inhibitory inputs; each I cell receives about 80 excitatory and 8 inhibitory inputs. All inputs have weight 1. The voltage of each neuron evolves as:

$$\begin{array}{ccc} C_{m}\frac{dV}{dt} & = & g_{L}\frac{\left(V-E_{L}\right)\left(V-E_{T}\right)}{E_{T}-E_{L}}-g_{\beta e}S_{\beta e}\left(\bar{V}-E_{syn}\right)-g_{\beta i}S_{\beta i}\left(\bar{V}-I_{syn}\right)\\ & - & g_{ad}z\left(\bar{V}-E_{K}\right)+I_{\beta }\left(t\right)+\zeta_{\beta }\Xi \left(t\right)\\ & \equiv & g_{L}\frac{\left(V-E_{L}\right)\left(V-E_{T}\right)}{E_{T}-E_{L}}+I_{total}\left(t\right) \end{array}$$ (11)

where *β* ∈ {*e*, *i*} and $\bar{V}=\left(E_{L}+E_{T}\right)/2$. *S*_*βe*_, *S*_*βi*_ are the weighted synaptic inputs. For example $S_{ee}=W_{E\to E}s_{e}$. *z* is spike frequency adaptation and is only applied to the excitatory cells, with conductance, *g*_*ad*_. A neuron spikes at time *t*_*sp*_ if $\text{lim}\;t\to t_{sp}^{-}V\left(t\right)=+\infty$ whence it is immediately reset to −∞. The synaptic variables, *s*_*e*_, *s*_*i*_ and the adaptation, *z* satisfy first order kinetics:

$$\frac{dw}{dt}=-w/\tau +\delta \left(t-t_{sp}\right)$$

where *w* ∈ {*s*_*e*_, *s*_*i*_, *z*}. Ξ(*t*) is Gaussian noise. Standard parameters are *C*_*m*_ = 1*μF*/*cm*^2^, *g*_*L*_ = 0.1*mS*/*cm*^2^, *g*_*ee*_ = 0.2*mS*/*cm*^2^, *g*_*ei*_ = 1*mS*/*cm*^2^, *g*_*ie*_ = 0.2*mS*/*cm*^2^, *g*_*ii*_ = 0.1*mS*/*cm*^2^, *E*_*L*_ = −65*mV*, *E*_*T*_ = −50*mV*, *g*_*ad*_ = 1*ms*/*cm*^2^, *E*_*K*_ = −85*mV*, *E*_*syn*_ = 0*mV*, *I*_*syn*_ = −75*mV*, $\zeta_{e}=\sqrt{0.05}$, $\zeta_{i}=0.5\sqrt{0.05}$, *τ*_*e*_ = 3*msec*, *τ*_*i*_ = 4*msec*, and *τ*_*z*_ = 50*msec*. In order to simulate this model exactly, we transform it to the theta model:

$$V=\bar{V}+\left(E_{T}-E_{L}\right)/2\;\text{tan}\left(\theta /2\right)$$

so that *θ* evolves as

$$\begin{array}{c} C_{m}\frac{d\theta }{dt}=-g_{L}\;\text{cos}\;\theta +\left(1+\text{cos}\;\theta \right)G_{total}\left(t\right), \end{array}$$ (12)

where *G*_*total*_(*t*) = 2*I*_*total*_(*t*)/(*E*_*T*_ − *E*_*L*_) is the input conductance. Spiking occurs when *θ* crosses *π* from below; *θ* is then reset to −*π* and the corresponding synaptic and adaptation variables are incremented by 1. We integrate the system of equations with Euler-Maruyama method using dt=0.05msec.

### Traveling waves

Traveling waves satisfy, *s*(*x*, *t*) = *S*(*x* + *ct*) = *S*(*ξ*) where *c* is the velocity of the wave, *ξ* = *x* + *ct* is the traveling coordinate, and *s* ∈ {*s*_*e*_, *s*_*i*_, *z*}. If the convolution kernels, *W*_*e*,*i*_(*ξ*) are exponential, then they can be inverted to become second order ODEs in *ξ*. This means that the system Eq (7) can be written as a 7-dimensional ODE (see Results for details) with a 2-dimensional unstable and 5-dimensional stable manifold. We use XPPAUT [46] to compute the homoclinic orbits for this system via continuation with AUTO (specifically HOMCONT, the homoclinic continuation package in AUTO). That is, we seek a traveling pulse solution to Eq (7), $U\left(\xi \right)=(s_{e}\left(\xi \right),s_{i}\left(\xi \right),s_{z}\left(\xi \right),S_{e}\left(\xi \right),S_{i}\left(\xi \right),S_{e}^{\prime }\left(\xi \right),S_{i}^{\prime }\left(\xi \right))$ such that *U*(*ξ*) tends to the equilibrium value, $\bar{U}$ as |*ξ*| → ∞. In order to use HOMCONT, we need a good starting approximation for the pulse. Since the stable manifold is two-dimensional, this is quite difficult numerically as two numbers must be simultaneously found. Instead, to get a starting approximation, we first consider the case where *σ*_*i*_ = 0 so that we have a 5-dimensional system. The equilibrium has a 1-dimensional unstable manifold which we approximate from the eigenvector of the positive eigenvalue. Given a guess for *c*, the wave velocity, we can compute the eigenvector, *V*^+^ corresponding to this unstable eigenvalue. We solve the five-dimensional system with $U\left(0\right)=\bar{U}\pm \epsilon V^{+}$ where *ϵ* is a small number (typically dt, the stepsize of our numerical integrator). We vary *c* until the trajectory returns close to $\bar{U}$ giving us a crude approximation to the homoclinic orbit. Next, we create a 7-dimensional system by appending the dynamics for *S*_*i*_ onto the 5-dimensional system. Using our crude approximation for the 5-dimensional system, we can vary $S_{i}\left(0\right),S_{i}^{\prime }\left(0\right)$ to get an approximation for the homoclinic in the 7-dimensional system. We refine this approximation in HOMCONT and then continue the homoclinic orbit in the parameters, *σ*_*i*_, *g*_*ei*_, and *g*_*ad*_.

### Step function approximation

We approximate the smooth nonlinearity *F* with a scaled Heaviside step function and use this to derive a series of nonlinear equations for the properties of the waves such as the velocity, onset of inhibition and the width of the excitatory and inhibitory pulses. (see Results) We use XPPAUT to study how these change with parameters since they are just roots of a set of nonlinear equations. We formally linearize about the traveling waves and compute the so-called Evans Function [26] (see results for details). We then use MATLAB to find the roots of the Evans Function in order to assess the stability of the waves. We use the fsolve function in MATLAB to find the curves of Hopf bifurcations.
